# Understanding Human Papillomavirus Vaccination Hesitancy in Japan Using Social Media: Content Analysis

**DOI:** 10.2196/68881

**Published:** 2025-02-11

**Authors:** Junyu Liu, Qian Niu, Momoko Nagai-Tanima, Tomoki Aoyama

**Affiliations:** 1 Kyoto University Kyoto Japan

**Keywords:** human papillomavirus, HPV, HPV vaccine, vaccine confidence, large language model, stance analysis, topic modeling

## Abstract

**Background:**

Despite the reinstatement of proactive human papillomavirus (HPV) vaccine recommendations in 2022, Japan continues to face persistently low HPV vaccination rates, which pose significant public health challenges. Misinformation, complacency, and accessibility issues have been identified as key factors undermining vaccine uptake.

**Objective:**

This study aims to examine the evolution of public attitudes toward HPV vaccination in Japan by analyzing social media content. Specifically, we investigate the role of misinformation, public health events, and cross-vaccine attitudes (eg, COVID-19 vaccines) in shaping vaccine hesitancy over time.

**Methods:**

We collected tweets related to the HPV vaccine from 2011 to 2021. Natural language processing techniques and large language models (LLMs) were used for stance analysis of the collected data. Time series analysis and latent Dirichlet allocation topic modeling were used to identify shifts in public sentiment and topic trends over the decade. Misinformation within opposed-stance tweets was detected using LLMs. Furthermore, we analyzed the relationship between attitudes toward HPV and COVID-19 vaccines through logic analysis.

**Results:**

Among the tested models, Gemini 1.0 pro (Google) achieved the highest accuracy (0.902) for stance analysis, improving to 0.968 with hyperparameter tuning. Time series analysis identified significant shifts in public stance in 2013, 2016, and 2020, corresponding to key public health events and policy changes. Topic modeling revealed that discussions around vaccine safety peaked in 2015 before declining, while topics concerning vaccine effectiveness exhibited an opposite trend. Misinformation in topic "Scientific Warnings and Public Health Risk" in the sopposed-stance tweets reached a peak of 2.84% (47/1656) in 2012 and stabilized at approximately 0.5% from 2014 onward. The volume of tweets using HPV vaccine experiences to argue stances on COVID-19 vaccines was significantly higher than the reverse.

**Conclusions:**

Based on observation on the public attitudes toward HPV vaccination from social media contents over 10 years, our findings highlight the need for targeted public health interventions to address vaccine hesitancy in Japan. Although vaccine confidence has increased slowly, sustained efforts are necessary to ensure long-term improvements. Addressing misinformation, reducing complacency, and enhancing vaccine accessibility are key strategies for improving vaccine uptake. Some evidence suggests that confidence in one vaccine may positively influence perceptions of other vaccines. This study also demonstrated the use of LLMs in providing a comprehensive understanding of public health attitudes. Future public health strategies can benefit from these insights by designing effective interventions to boost vaccine confidence and uptake.

## Introduction

Cervical cancer remains a significant public health challenge worldwide. In 2020, the World Health Organization (WHO) launched a strategy to eliminate cervical cancer, aiming to achieve 90% coverage rate for human papillomavirus (HPV) vaccination by 2030 [1]. In 2023, the WHO released estimates of first-dose vaccination coverage for women in 133 countries, with a global average of 62%, and 15 countries have already met the WHO’s target [2]. However, Japan presents a unique case among high-income countries, where HPV vaccination rates have remained critically low. Both bivalent and quadrivalent HPV vaccines were licensed and subsequently included in the national immunization program for girls in 2013 in Japan [3,4]. However, widespread reports of adverse events prompted the Japanese Ministry of Health, Labour and Welfare (MHLW) to suspend the proactive recommendation in 2013, leading to a sharp decline in public trust and a significant increase in cervical cancer incidence [5-13]. While proactive recommendations were reinstated in 2022, HPV vaccine uptake remains low [14-18].

To enhance HPV vaccination uptake in Japan, it is crucial to systematically analyze the trends of vaccine hesitancy. Existing literature reveals varying levels of hesitancy across different populations. Studies targeting parents consistently demonstrated significant hesitancy in the years preceding the updated recommendations, whereas surveys involving health care professionals, including those who are parents, showed higher confidence. A 2024 MHLW survey found comparable proportions of willingness, unwillingness, and uncertainty among both the target population and their parents. However, a critical gap in current research is the lack of longitudinal studies to track public attitudes toward HPV vaccination over time using consistent methodologies.

Understanding the factors influencing vaccine hesitancy is also essential. Studies have attributed this to persistent public distrust, misinformation, negative media coverage [15-20], and prolonged government suspension of recommendations, which heightened public concern [11]. The WHO Global Advisory Committee on Vaccine Safety emphasized the potential harm of policy decisions based on weak evidence, leading to the underutilization of safe and effective vaccines [21]. However, limited research has comprehensively investigated the long-term, dynamic influence of the key events and factors on HPV vaccine hesitancy in Japan.

In recent years, social media platforms have emerged as valuable resources for public health research. Comparing with the traditional survey research, social media can provide large volumes of real-time data that are readily accessible and searchable [22]. Among all the social media platforms, the user base of Twitter (subsequently rebranded as X) in Japan has grown significantly, with its share of users increasing from slightly more than 20% of the population in 2014 to approximately 45% in 2021 [23]. The platform’s primary active user demographic, aged 13-39 years, substantially overlaps with the target age groups of vaccine-eligible adolescents and their parents, making it a relevant data source for analyzing public discourse on the HPV vaccine [24]. Twitter analysis has been applied to investigate public discourse surrounding HPV vaccination in various countries [25-28]. In Japan, Suzuki et al [29] analyzed 208 geotagged tweets, revealing an uneven distribution of users disseminating information related to HPV vaccination. Terada et al [19] examining 3623 tweets collected over a 7-month period, reported a predominance of negative sentiment associated with discussions pertaining to HPV vaccine safety and side effects. However, few works used large-scale social media analysis to comprehensively examine the evolution of public discourse concerning HPV vaccination over an extended period within the Japanese context.

Recent advancements in natural language processing (NLP) methodologies, particularly the integration of deep learning (DL) models, have significantly enhanced the accuracy and sophistication of social media analysis [30]. While traditional NLP techniques such as n-grams [31] and topic modeling [32] have proven valuable, DL models, especially the large language models (LLMs), have demonstrated significant potential for conducting sophisticated and nuanced analyses of social media data [33]. However, the application of LLMs to investigate HPV vaccine hesitancy in Japan remains an underexplored area of research.

This study aims to address these gaps by analyzing the long-term evolution of public attitudes toward HPV vaccination in Japan from 2011 to 2021 through Twitter data. Specifically, we use advanced NLP techniques, including stance analysis, time series analysis, topic modeling, and logic analysis, to:

1. Examine the dynamics of HPV vaccine confidence over time;
2. Identify changes in key factors influencing vaccine hesitancy; and
3. Explore the relationship between key events and shifts in public opinion, including interaction between vaccines.

By leveraging state-of-the-art NLP and LLM methods, this study provides a systematic, data-driven understanding of vaccine hesitancy in Japan. The findings will offer insights into the evolution of public perceptions and inform strategies to address vaccine hesitancy more effectively.

## Methods

### Data Collection and Preprocessing

This study used the Twitter application programming interface to collect Japanese tweets related to HPV vaccination between 2011 and 2021. The tweets were retrieved using various Japanese keywords for HPV vaccine. The number of tweets increased each year, resulting in 228,376 tweets. After excluding tweets with unrecognizable dates, 228,300 were retained for subsequent analyses. After data collection, the tweets were cleaned and preprocessed. Retweets were removed using the Python package *Tweepy* (Twitter) [34]. Web links, special characters, emojis, and ampersands were eliminated, and full-width English characters were converted into half-width lowercase characters.

### Annotation

To facilitate model training and fine-tuning, 2.5% of the tweets per year were randomly selected for annotation. This study focused on public stance toward the HPV vaccine by using a 3-category annotation system: advocate, oppose, and unknown. The detailed category definitions are shown in Table 1.

**Table 1:** Definitions of stances to the human papillomavirus vaccine.

Category	Definition	Mock tweet example
Advocate	Affirmatively advocacy of the HPV^a^ vaccine, highlighting its efficacy in preventing HPV-related diseases, the critical role of vaccination in public health, and positive vaccination outcomes. These messages distinctly advocate for the HPV vaccine’s utilization, reinforcing its importance in health prevention measures.	Just got my HPV vaccine today! Feeling grateful for the science that protects us from cervical cancer. Highly recommend it to everyone eligible!
Opposite	Explicitly challenge or critique the HPV vaccine, focusing on concerns related to its efficacy, safety, or potential adverse effects. The critical perspective must be distinctly directed at the HPV vaccine in isolation, excluding broader criticisms of vaccines at large, political deliberations concerning vaccination policies, or financial considerations associated with vaccine procurement.	I’m skeptical about the HPV vaccine due to the side effects I’ve read about. More research is needed before making it a widespread recommendation. Let’s be cautious.
Unknown	Neither explicitly endorse nor directly oppose the HPV vaccine. This category encapsulates tweets that voice opposition stemming from broad antivaxxers, policies, or concerns about the cost, without specifically expressing the advocate or opposite of the HPV vaccine itself. It also includes tweets that are too ambiguous, neutral, or irrelevant to ascertain a clear position concerning the HPV vaccine.	Why isn’t the HPV vaccine free for everyone? If it’s so important, shouldn’t the government cover the cost? Still trying to understand the policy behind this.

^a^HPV: human papillomavirus.

The annotation team comprised 4 medical professionals, 3 of whom independently annotated each tweet. Tweets with unanimous annotations were classified as tier 1, those with 2 matching annotations as tier 2, and those without matching annotations as tier 3. Tier 1 data, which were deemed to have the highest consistency, used the initial annotations as the final labels. Tier 2 and 3 data underwent further consensus meetings, with the final labels determined by the last medical professional. A total of 5758 tweets were annotated, with 5050 in tier 1, a total of 701 in tier 2, and 7 in tier 3. The Cohen κ values between each pair of the 3 major medical professionals (KG, YN, and ND) are shown in Table 2, which are all over 0.8 and at the agreement level of almost perfect. The distributions across the advocate, oppose, and unknown categories in the annotated data were 1646, 697, and 3415, respectively.

**Table 2:** Cohen κ between the annotations by each pair of medical professionals.

	Cohen κ
KG and YN	0.851
YN and ND	0.845
KG and ND	0.858

### Stance Analysis With DL Models

#### Model Selection

To achieve the optimal results, we first selected the best-performing model. We compared several traditional NLP classification models with LLMs. Among the traditional models, we considered long short-term memory [35], Bidirectional Encoder Representations from Transformers (BERT) [36], and DistilBERT [37]. For the LLMs, we used Gemma-2-2b [38], Gemma-2-9b, and Llama-3.1-8b [39]. All uses of open-source LLMs in this work were conducted following approval and in strict accordance with their respective licensing agreements. We also evaluated the Gemini Pro 1.0 [40] using Google AI Studio during the free trial.

A 2-layer bidirectional architecture is designed for the long short-term memory model. The tweets were tokenized using the *Ginza* Python package (Megagon Labs) [41]. Owing to the manageable parameter size of this model, it was trained from scratch. For the BERT models, Tohoku BERT [42] and Line DistilBERT [43] were selected as baselines. A classification head was added to the first token of the final hidden layer of these models, and fine-tuning was performed using our tweet data, while keeping the BERT parameters frozen.

For LLMs, computational resource constraints prevented us from fine-tuning models with more than 10 billion parameters, whereas budgetary constraints precluded fine-tuning using application programming interfaces such as ChatGPT [44] or Claude [45]. Therefore, we opted for smaller open-source LLMs and tested both baseline, Quantization, and Low-Rank Adaptation fine-tuned versions on the tweet data [46]. For Gemini Pro 1.0, we fine-tuned the model directly using Google AI Studio. For all LLMs, we used the following prompt in Japanese, followed by the original tweet, as user prompts:

Does this tweet express a position against the HPV vaccine itself? Please classify this tweet as “advocate,” “oppose,” or “unknown.” Do not make subjective assumptions; base your decision solely on the content of the tweet. Answer only your position, and no explanation is required.

Given the significant class imbalance in the dataset, 5758 annotated tweets were split into training, validation, and test sets, with the proportion of each category maintained across the splits. To ensure a robust evaluation, 100 tweets from each category were randomly selected to form a test set. The remaining data within each category were partitioned into training and validation sets at a ratio of 4:1. Due to the training sample size limitation (<500) imposed by Gemini 1.0 Pro, a subset of 498 labeled tweets (distributed equally across the 3 categories at a ratio of 166:166:166) was used to fine-tune the model for the HPV vaccine stance classification task. The model performance was assessed using standard evaluation metrics, namely, precision, recall, and *F*_1_-score. The details of the labeled dataset are listed in Table 3.

**Table 3:** Number of labeled tweets of different stances in the dataset.

Category	Train set, n	Validation set, n	Test set, n	Total, n
Advocate	2652	663	100	3415
Oppose	477	120	100	697
Unknown	1236	310	100	1646
Total	4365	1093	300	5758

To investigate the performance boundaries of the optimal model further, we explored the impact of varying category distributions within the training data. We experimented with different ratios of advocate, opposition, and unknown categories, specifically 166:166:166, 150:200:150, 125:250:125, 100:300:100, 75:350:75, and 50:400:50. For each ratio, the data were randomly sampled 3 times, the model performance was evaluated, and the results were averaged to ensure robustness.

After identifying the optimal category ratio, we fine-tuned the best-performing model 5 times using this ratio, each time with a new random data sample. The remaining labeled data were used for the performance evaluation, and the 3 models exhibiting the highest performance were selected for the final inference. For each unlabeled tweet, the 3 models generated independent predictions. If 2 or more models agreed with the predicted label, they were assigned to the tweet. When all 3 models produced different predictions, the tweet was classified as “unknown.”

#### Time Series Analysis of Stances

Following the stance classification of all tweets, a comprehensive time series analysis was performed on the extracted data to examine the evolution of public sentiments toward HPV vaccines in Japan between 2011 and 2021. The HPV vaccine supply and injection data were collected from the official website of MHLW. The raw data for vaccine supply lack fine-grained annual data and can be across 2 years, so the annual supply for some years were based on estimation; details are provided in Multimedia Appendix 1. We calculated the annual advocate rate from the tweets by *(number of advocate tweets)/(number of advocate tweets + number of opposed tweets)*. The difference between the monthly counts of tweets advocating for and opposing the HPV vaccine was calculated to capture the dynamic shifts and relative strength of each stance over time.

To identify significant structural breaks within the time series data, the pruned exact linear time (PELT) algorithm [47], a computationally efficient method for change-point detection, was used. These identified change points correspond to major shifts in public opinion regarding the HPV vaccine, potentially linked to specific events or information dissemination campaigns. By juxtaposing the time series analysis results with a timeline of key news events and public health policies, a comprehensive interpretation of the observed trends was achieved. This approach facilitates a deeper understanding of the factors influencing the public acceptance of HPV vaccines and provides empirical evidence for future public health interventions and policy decisions.

### Latent Dirichlet Allocation Topic Modeling Analysis

In this study, we used latent Dirichlet allocation (LDA) [32] topic modeling, a widely used technique for extracting latent thematic information from large volumes of text data, to analyze Japanese tweets related to the HPV vaccine between 2011 and 2021. Using LDA modeling, we aimed to gain a deeper understanding of the specific perspectives held by Japanese Twitter users across different stances toward the HPV vaccine. To determine the optimal number of topics that best represented the thematic structure of the dataset, we experimented with various topic numbers, ranging from 1 to 50. For each configuration, the performance of the model was evaluated using perplexity and coherence scores [48]. Details of the optimal topic number selection are shown in Figures S1, S4, and S7 in Multimedia Appendix 2. After the optimal number of topics was selected, qualitative analyses were conducted to refine and interpret the final thematic content. Similar to Niu et al [49], we calculated the monthly average expectation of tweets belonging to different topics for a fine-grain time series analysis.

In line with prior research indicating that misinformation is an important factor in vaccine hesitation [19,50,51], we also investigated the impact of misinformation on the HPV vaccine. First, we identified topics that were likely to contain misinformation through group discussion, focusing on tweets with an estimated probability of more than 80% of belonging to those topics. We then used the Claude-3-opus model [45] to assess whether each tweet attempted to disseminate misinformation regarding the HPV vaccine. To validate the accuracy of the model’s classification, we randomly selected 25 tweets each categorized as misinformation and 25 categorized as non-misinformation by the model. Three independent volunteers evaluated the credibility of the information in these tweets by cross-referencing reputable news sources and data from the MHLW [52]. The verification process was repeated 3 times to estimate the classification accuracy of the model.

### Explore Relationship Between HPV Vaccination and COVID-19

Considering the chronological overlap between the duration of data collection and the COVID-19 pandemic, which is a significant historical health event, this study investigated the potential relationship between HPV vaccination and COVID-19. Specifically, the objective of this study was to determine whether the COVID-19 pandemic had an impact on public attitudes toward HPV vaccination. From our dataset, all tweets comprising the keyword “COVID-19” in both Japanese and English were extracted. In addition to the time series analysis and LDA topic modeling previously described, the correlation between all HPV vaccine–related tweets and the tweets incorporating the “COVID-19” keywords was examined. Moreover, specific time points where the total number of tweets and the tweets containing “COVID-19” keywords exhibited simultaneous increases were investigated. To estimate the percentage of tweets pertaining to pivotal events, 100 tweets were randomly selected 3 times at each time point. Three volunteers verified the number of tweets related to the paramount event, and the percentage of related tweets was calculated by averaging the 3 results and computing CIs. To gain a deeper understanding of the data spikes, the influence of COVID-19 vaccine–related pivotal events on peak time points was also assessed.

Furthermore, we investigated the potential causality between the stance toward the HPV vaccine and the stance toward COVID-19. Specifically, we analyzed tweets containing “COVID-19” keywords and expressing either an advocate or opposed stance. We used logic analysis by LLM to determine whether the tweet used the HPV vaccine as an illustrative example to convey the author’s perspective on COVID-19 and vice versa. The Claude-3-opus model was directly used for inference, with the prompt instructing it to read the tweet and provide an output of “HPV to COVID” if the tweet used the HPV vaccine as an example to express an idea about the COVID-19 vaccine, “COVID to HPV” if the reverse was true, or “Not related” if neither was applicable. The classified tweets were tallied weekly and visually presented to demonstrate the results.

The manuscript was prepared in accordance with the iCHECK-DH: Guidelines and Checklist for the Reporting on Digital Health Implementations [53]. The IDs of the tweets used in this research are provided in Multimedia Appendix 3.

### Ethical Considerations

This study used publicly available and accessible tweets. We assert that our analysis is compliant with Twitter’s usage policy in aggregate form without identifying specific individuals who published the Twitter posts. Furthermore, the number of vaccination supply and injection downloaded from the MHLW are open government data. Therefore, the activities described do not meet the requirements of human subject research and did not require review by an institutional review board.

## Results

### Model Selection

A comparison of all the models tested is shown in Multimedia Appendix 4. The *F*_1_-scores of the fine-tuned larger models achieved better performance in most cases, except for Llama 3.1. This may be because it was not instruction tuned for Japanese corpora in advance. The fine-tuned Gemini 1.0 pro yielded the best results in our experiment, and we decided to perform the following steps based on Gemini 1.0 pro. Detailed results are shown in Multimedia Appendices 5 and 6.

Because the allowed number of tweets for fine-tuning Gemini 1.0 pro was fixed (<500), we experimented with different data ratios to determine the most effective category ratio to fine-tune the model. The results indicated that the model performed best when the ratio of the advocate, opposite, and unknown categories was 150:200:150. This ratio was consistent in the validation experiments, achieving the highest average *F*_1_-score of 0.968, as shown in Multimedia Appendix 7. The detailed model performance for each ratio is shown in Multimedia Appendix 8.

After 5 rounds of random data selection and fine-tuning using the optimal data ratio, the performance of the fine-tuned Gemini 1.0 Pro model was evaluated using the remaining labeled data. The evaluation results showed significant improvements in precision, recall, and *F*_1_-score for the fine-tuned model. The average *F*_1_-score of the 3 best-performing models was 0.924, with a precision of 0.928 and a recall of 0.924. In comparison, the baseline performance of the untuned Gemini 1.0 Pro model on the same test dataset showed an *F*_1_-score of 0.781, a precision of 0.829, and a recall of 0.796. This comparison clearly demonstrates the importance of fine-tuning for enhancing the performance of the model for specific tasks.

When classifying the unlabeled Japanese tweets, the results from the 3 best models were mostly consistent. Among all tweets, 86.85% (198276) received 3 identical result labels and 13.06% (29806) received 2 identical result labels. In cases where all 3 labels were completely inconsistent, these tweets were classified as unknown, accounting for 0.09% (209) of all the tweets.

### Time Series Analysis of Stance

**Figure 1:**
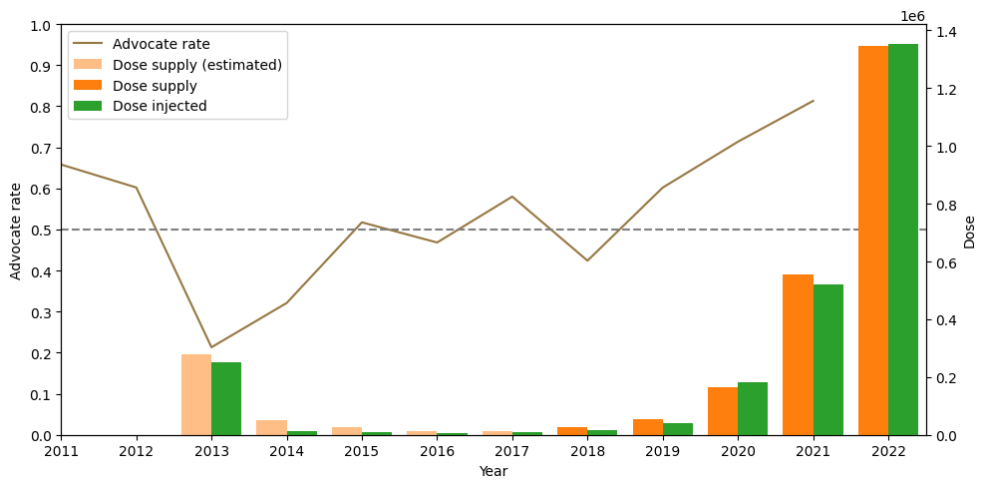
Advocate rate toward human papillomavirus vaccine between 2011 and 2021 (line) and the number of vaccine supply and injection between 2013 and 2022 (bars). The vaccine supply from 2013 to 2017 was estimated based on Japanese Ministry of Health, Labour and Welfare (MHLW) official data due to lacking fine-grained annual data, and the vaccine supply after 2017 is from MHLW official data.

We first analyzed the trend of advocate rate toward the HPV vaccine from 2011 to 2021 and the HPV vaccine supply, injection from 2013 to 2022, as shown in Figure 1. It is noticeable that vaccine supply between 2013 and 2017 is based on estimation because of lacking fine-grained annual data. The lowest advocate rate appeared in 2013, when the Japanese government decided to suspend its recommendations for the HPV vaccine, which was announced only 2 months ago [54], leading to a sharp decrease of vaccine supply and injection 2014. The advocate rate recovered in 2 years and stayed to be around 50% until 2018, but the vaccine supply and injection stayed at an ignorable level. The advocate rate increased and stayed in a high level since 2019, during which 2020 witnessed numerous petitions urging the government to reinstate active recommendations and prompt policy reviews [55]. The vaccine supply and injection started exponential growth since 2019, especially after the proactive HPV vaccine recommendations reinstated in 2022 [14-18]. Vaccine injected surpassed vaccine supply in 2020 and 2022, indicating that the vaccines remained last year were consumed.

**Figure 2:**
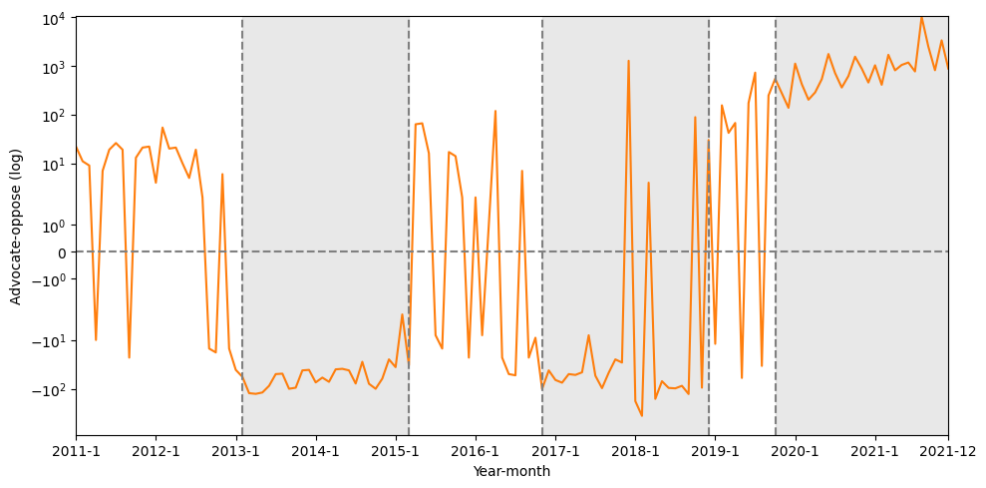
Monthly differences between number of tweets of advocate and oppose stances. The significant low and high periods based on change points detected by pruned exact linear time are marked in gray.

A time series analysis of the difference between advocates and opposed stances is shown in Figure 2, which shows several pivotal junctures marked by substantial shifts in the disparity between pro- and anti-HPV vaccination stances, suggesting notable fluctuations in public opinion. Overall, we can observe that there are 3 periods of fluctuation, and the 3 periods are relatively stable in stances. Using the PELT algorithm, we successfully discerned structural change points within the data, closely aligning with significant shifts in public attitudes toward HPV vaccines. Notably, these change points were observed in 2013, 2016, and 2020, coinciding with key public health events and policy developments. Beside the events in 2013 and 2020, in late 2016, widespread discourse surrounding vaccine safety was triggered by legal actions taken by individuals alleging adverse side effects [56].

### LDA Topic Modeling Analysis

We separately applied LDA modeling to the tweets of advocates, opposes, and unknown stances. There are 3 best topic numbers for advocates and unknowns and 4 for opposition. Details of the top words and weights are provided in Multimedia Appendix 2. We then calculated the ratio of the monthly expectations of tweets belonging to different topics to obtain fine-grained insights into the weights of the topics in each stance.

**Figure 3:**
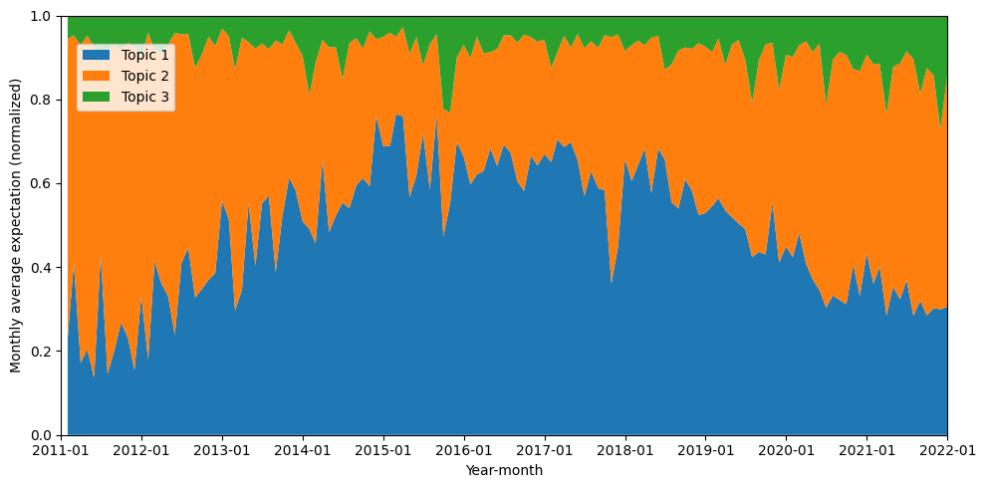
Monthly change of ratio of different topics in tweets of advocate stance from 2011 to 2021.

The distribution of various themes within the “advocate” stance is illustrated in Figure 3. The proportion of “Scientific and Media Discourse on HPV Vaccine Safety” (topic 1) underwent a significant increase until 2015, subsequently experiencing a gradual decline. Conversely, the proportion of “HPV Vaccine Effectiveness and Broader Public Health Measures” (topic 2) diminished, reaching its lowest point around 2015, after which it exhibited a consistent increase. The proportion of “Policy and Advocacy for HPV Vaccination Promotion” (topic 3) remained relatively stable, although a slight upward trend has been observed since 2019.

**Figure 4:**
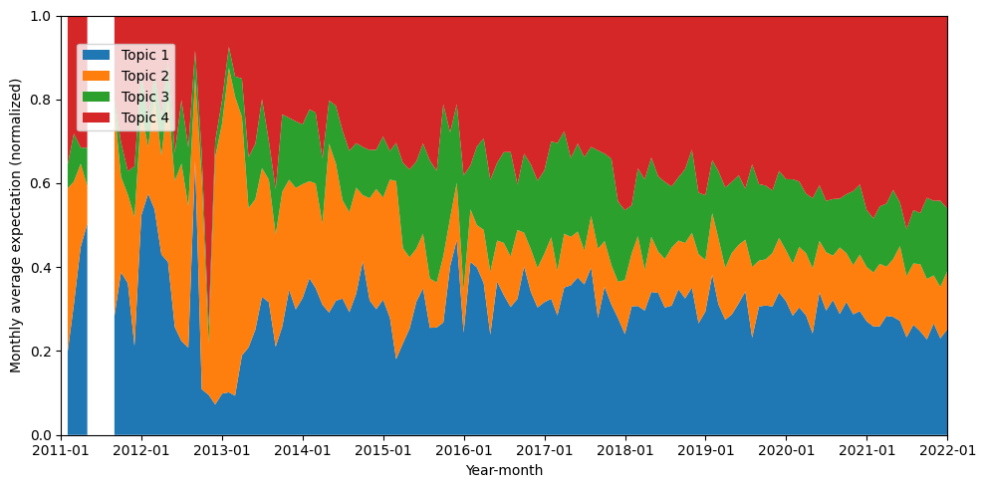
Monthly change of ratio of different topics in tweets of opposed stance from 2011 to 2021.

In the analysis of tweets expressing the “opposing” stance in Figure 4, “Skepticism and Opposition to Vaccination” (topic 2) demonstrated notable peaks in weight during the years 2013 and 2015, followed by a gradual decline and eventual stabilization of topic weight. The proportion of “Scientific Warnings and Public Health Risks” (topic 4) surged in late 2012, followed by a sustained increase throughout the decade, ultimately becoming the dominant theme within an opposing stance.

**Figure 5:**
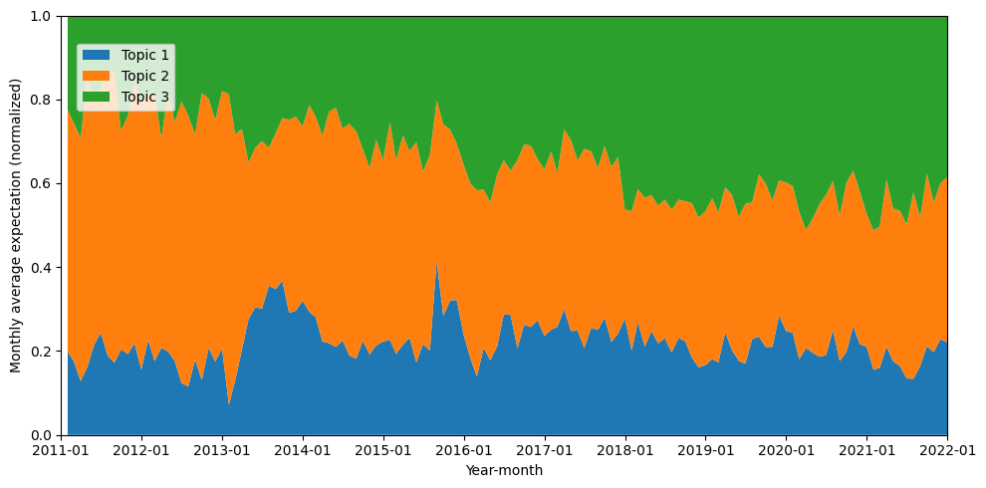
Monthly change of ratio of different topics in tweets where the stances are unknown from 2011 to 2021.

In the tweets classified as “unknown” by stance shown in Figure 5, “HPV Vaccine Efficacy and Public Health Initiatives” (topic 2) was the dominant theme prior to the year 2013 and then declined thereafter. Conversely, the proportion of tweets expressing “Opposition, Misinformation, and Activism Surrounding HPV Vaccination” (topic 3) displayed a gradual increase and has become the predominant theme since 2018. Notably, the theme of “HPV Vaccine Safety and Governmental Oversight” (topic 1) exhibited a gradual increase throughout the entire period under consideration.

**Figure 6:**
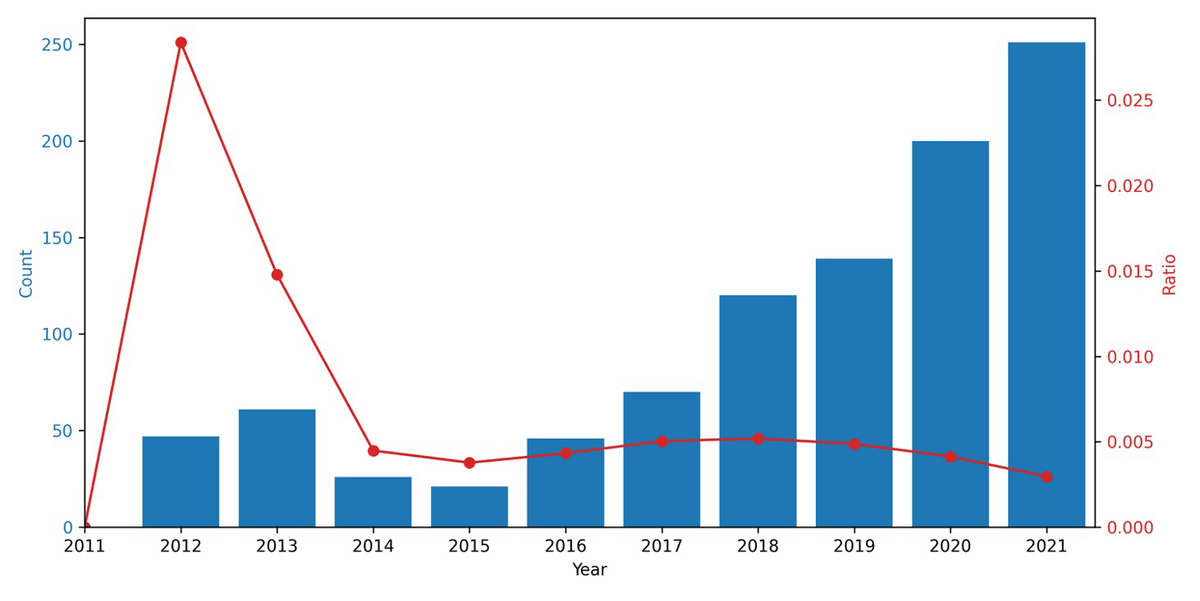
Count and ratio of tweets classified as misinformation. The bars show the total number of tweets detected as misinformation each year, and the line shows the ratio of the tweets containing misinformation within the total number of tweets of opposed stance each year.

Then, we applied misinformation detection to certain topics. In tweets of opposed stance, we identified “Scientific Warnings and Public Health Risks” (topic 4) as the most likely to contain misinformation about the HPV vaccine. We extracted tweets with a probability of more than 80% belonging to this topic, yielding 3001 tweets. The misinformation classification results obtained using Claude-3-opus are shown in Figure 6. The classification accuracy, determined through random sampling and verification conducted thrice, ranged from 95% CI 89.74% to 100%. Our findings revealed that the prevalence of misinformation increased in most years, except for 2014 and 2015. The ratio of misinformation in HPV vaccine–related tweets began to spike sharply in 2012, followed by a rapid decline to its nadir in 2015. Subsequently, the ratio gradually increased, reaching a secondary, albeit lower, peak in 2018, after which it began to decline gradually.

### Relationship Between HPV Vaccination and COVID-19

**Figure 7:**
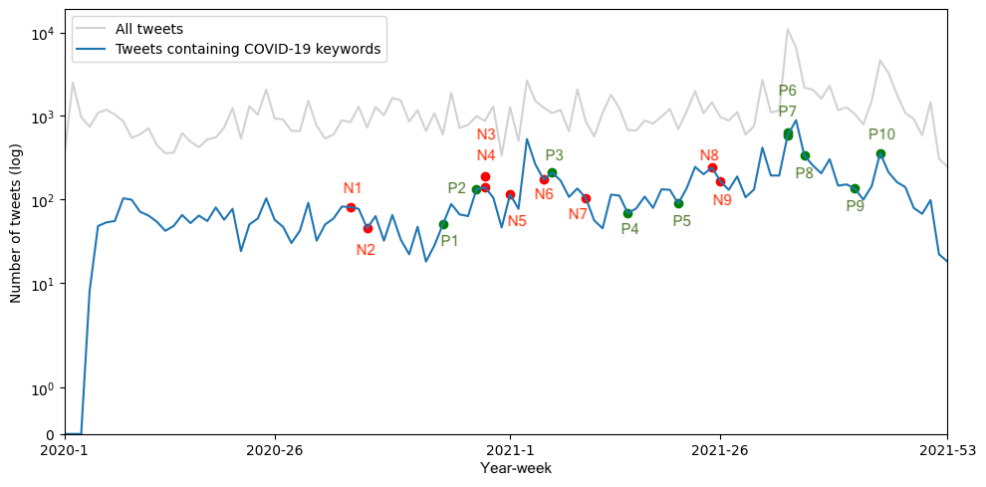
Number of all tweets related to human papillomavirus vaccine (gray), and the tweets containing the “COVID-19” keywords (blue), with key events related to COVID-19 vaccine marked. The points beginning with “N” are negative key events that may lead to opposition to COVID-19 vaccines, and the points beginning with “P” are positive key events that may lead to advocacy to COVID-19 vaccines.

We conducted a weekly time series analysis of tweets containing COVID-19 keywords, as illustrated in Figure 7. There are no tweets with “COVID-19” keyword in the first 2 weeks of 2020, so all the calculations began from the third week. The Pearson correlation coefficient (*r*) was 0.793 (*P*=1.59×10^–23^), indicating a relatively high correlation between all tweets and those containing “COVID-19” keywords. The time lag for the highest cross-correlation was zero. Several peaks in both all tweets and tweets containing “COVID-19” keywords were observed in 2021 (week 3, weeks 34-35, and week 45), with the 34th-35th weeks exhibiting the highest peak during the entire study period, which coincided with a campaign to call for resumption of active HPV vaccination recommendations [57] (95% CI 37.0%-39.8% HPV vaccine-related tweets; 95% CI 1.5%-9.1% tweets with “COVID-19” keywords).

**Figure 8:**
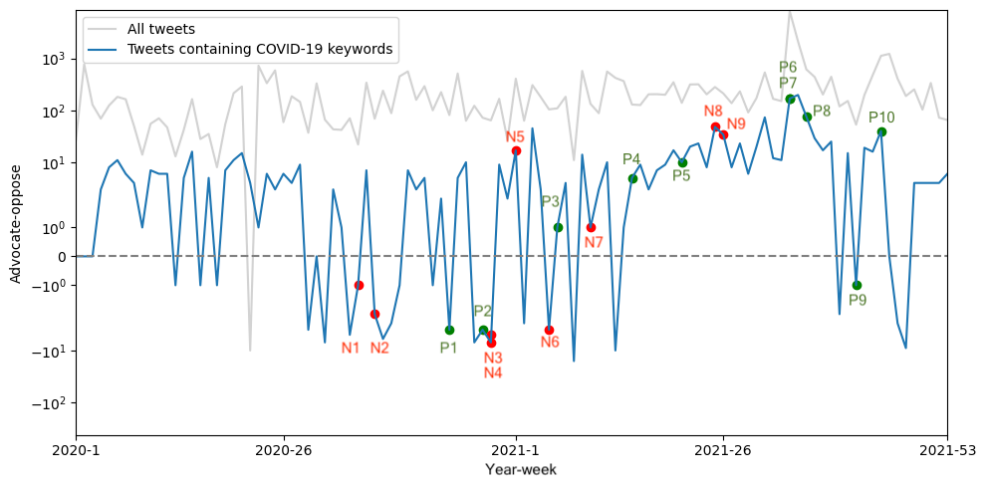
The difference between tweets of advocate stance and oppose stance for all human papillomavirus vaccine tweets (gray) and tweets containing the “COVID-19” keyword (blue), with key events related to COVID-19 vaccine marked. The points beginning with “N” are negative key events that may lead to opposition to COVID-19 vaccines, and the points beginning with “P” are positive key events that may lead to advocacy to COVID-19 vaccines.

A comparative analysis was performed to examine the differences between advocate and opposed stances in both the overall tweet corpus and the subset containing the “COVID-19” keyword, as illustrated in Figure 8. The *r* between advocate and oppose sentiment differences is substantial (*r*=0.723; *P*=6.09×10^–18^), with no temporal lag observed in the highest cross-correlation. In the broader context of all HPV vaccine–related tweets, advocacy sentiments generally prevailed over opposing sentiments. However, for tweets containing “COVID-19” keywords, the dominant stance fluctuated over time. Both datasets exhibited a pronounced peak in advocacy during weeks 34-35 of 2021, whereas opposing sentiments dominated in week 22 of 2020 for all tweets.

We additionally assessed the impact of COVID-19 vaccine–related events on the volume of all HPV vaccine–related tweets and tweets containing the keywords “COVID-19.” Details of the key events were gathered from Japan Broadcasting Corporation (NHK) [58] and are provided in Table S1 in Multimedia Appendix 2. As illustrated in Figure 7, it is evident that the key events do not consistently result in peaks in tweet volume for both all HPV vaccine–related tweets and tweets containing the keywords “COVID-19.” Furthermore, as depicted in Figure 8, positive and negative events do not always lead to an increase in the number of supportive or opposing stances.

LDA was conducted on tweets containing COVID-19 keywords advocating for and opposing stances. Multimedia Appendix 2 shows detailed results. To investigate nuanced changes in topic weights over time, the expectation of tweets belonging to different topics for both advocate and opposition stances was calculated, as shown in Multimedia Appendix 2. Analysis of these data revealed no significant changes in the proportions of different topics between 2020 and 2021.

**Figure 9:**
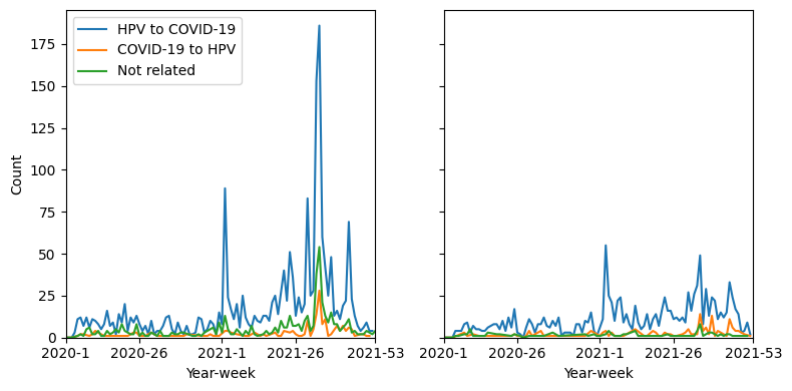
Weekly number of tweets with “COVID-19” keywords, using HPV vaccine as example to share stance to COVID-19 vaccine (“HPV to COVID-19”), using COVID-19 vaccine as example to share stance to HPV vaccine (“COVID-19 to HPV”), or neither (“Not related”) in 2021. (A) Result on tweets with advocate stance. (B) Result on tweets with opposed stance. HPV: human papillomavirus.

Finally, we investigated the potential causal relationship between attitudes toward HPV and COVID-19 vaccines using logical analysis. To evaluate the accuracy of Claude-3-opus in performing the classification task without fine-tuning, we randomly sampled 100 tweets 3 times and allowed 3 volunteers to judge the correctness. The accuracy is 95% CI 72.92%-91.74%. The weekly classification results are shown in Figure 9. The “HPV to COVID-19” category predominated throughout the entire study period for both supportive and opposing stances. Notably, tweets advocating for vaccines consistently outnumbered those opposing vaccines across all categories.

## Discussion

### Principal Findings

In this study, we used traditional NLP models and LLMs to perform a stance analysis of social media content, focusing on the public discourse around the HPV vaccine. Our findings demonstrate that LLMs provide a more nuanced understanding of public attitudes than traditional sentiment analysis, revealing critical insights into factors influencing vaccine hesitancy, such as confidence, complacency, and convenience. Specifically, we observed a slight increase in public advocacy and vaccine confidence toward HPV vaccines over time, influenced by policy decisions, media coverage, and public communication efforts. The WHO’s 3Cs model (Confidence, Complacency, and Convenience) proved instrumental in framing these dynamics and offers a pathway for policy makers to address vaccine hesitancy in Japan. We also observed some evidence of the positive influence of stance toward HPV vaccine on the stance toward COVID-19 vaccine.

Confidence pertains to trust in the vaccine’s safety, efficacy, and the health care system responsible for its administration and plays a key role in the stance toward the HPV vaccine in Japan. In our analysis, we observed significant fluctuations in the public stance toward the HPV vaccine, which were closely linked to government policy decisions and media coverage. The 2013 suspension of the HPV vaccine recommendations by the Japanese government was a key moment that led to a sharp decline in public trust. This decline can be attributed to increased public uncertainty and fear regarding vaccine safety, as shown in the time series analysis. The LDA topic modeling results also support this, showing a decline in the proportion of tweets related to “HPV Vaccine Efficacy and Public Health Initiatives” (topic 2) where the stance is unknown after 2013, while “Opposition, Misinformation, and Activism Surrounding HPV Vaccination” (topic 3) gradually became the predominant theme since 2018, highlighting growing public distrust and misinformation. Conversely, the resurgence of advocacy in 2020 reflected an improvement in public confidence, which likely influenced efforts to reinstate vaccine recommendations. This is directly shown by the exponential increase in vaccine supply and vaccine uptake and also reflected in the increased proportion of tweets related to “Scientific and Media Discourse on HPV Vaccine Safety” within the advocacy stance, which showed a significant increase until 2015, followed by a gradual decline, indicating ongoing advocacy efforts. Our findings illustrate that confidence is highly sensitive to policy decisions and that restoring public trust after a decline requires consistent and clear communication.

Furthermore, we explored the role of misinformation in undermining public confidence regarding HPV vaccinations. The spike in the ratio of tweets containing misinformation in 2012 likely contributed to the significant drop in vaccine confidence observed the following year, which aligns with findings from other studies [59-61]. From 2014 onward, the ratio of misinformation exhibited slight changes but remained consistent. The overall number of misinformation tweets increased in most years, indicating the persistent and long-lasting impact of misinformation. Therefore, addressing misinformation is essential for restoring and maintaining public trust in vaccines, because unchecked misinformation can severely erode public confidence and contribute to vaccine hesitancy.

Complacency refers to the perceived necessity of vaccination, which diminishes when individuals consider the risk of vaccine-preventable diseases low. Adjei Boakye et al [62] found that “not necessary” was consistently among the top reasons cited by parents for not vaccinating their children against HPV between 2010 and 2020. The topic “HPV Vaccine Efficacy and Public Health Initiatives” under the “unknown” stance experienced a sharp decline post-2013, indicating diminished public engagement and perceived necessity. The perceived low risk of HPV, combined with concerns about its side effects, may contribute to a reduced sense of urgency regarding vaccination. However, our time series analysis showed that complacency began to decrease in 2020 as advocacy efforts intensified, and the public became more aware of the risks of HPV and the benefits of vaccination. The upward trend in the topic “HPV Vaccine Effectiveness and Broader Public Health Measures” within the “advocate” stance since 2015 further reflects this shift toward increased awareness and decreasing complacency. This shift underscores the importance of proactive communication regarding the risk of vaccine-preventable diseases to counteract complacency.

Convenience encompasses factors such as availability, affordability, and accessibility of vaccination. In our study, the importance of convenience became evident when analyzing the periods of increased advocacy for HPV vaccination. The vaccine doses injected surpassed the doses supplied in 2020 and 2022, indicating that the inject number may be limited by the supply. Also, the resurgence of advocacy in 2020 coincided with governmental initiatives aimed at improving access to the HPV vaccine, including the reintroduction of public recommendations and enhancement of vaccination services. This alignment is reflected in the relatively stable proportion of tweets related to the topic “Policy and Advocacy for HPV Vaccination Promotion” within the advocacy stance, which exhibited a slight upward trend since 2019. These changes may encourage efforts to facilitate access to vaccination services and play a critical role in influencing public willingness to get vaccinated over time.

In addition to the findings regarding the HPV vaccine, we also noticed that stances toward the HPV vaccine may influence stances toward the COVID-19 vaccine during the COVID-19 pandemic. Specifically, our logical analysis revealed that increased confidence in the HPV vaccine might lead to higher confidence in the COVID-19 vaccine. Tweets categorized under “HPV to COVID-19”—which used the HPV vaccine as a reference point for the COVID-19 vaccine stance—predominated throughout the study period for both supportive and opposing stances. Notably, vaccine advocacy consistently outnumbered opposition across all categories, suggesting that rising confidence in one vaccine may positively affect confidence in others.

The findings of this study provide actionable insights for public health policies and interventions in Japan. First, improving confidence in vaccines requires transparent and consistent communication from government bodies and health care professionals, particularly during crises. Proactively addressing misinformation through targeted campaigns on social media can help rebuild public trust. To tackle complacency, educational programs highlighting the risks of HPV and the benefits of vaccination should be prioritized. Finally, enhancing convenience by ensuring vaccine availability, affordability, and accessibility—such as expanding vaccination services and public recommendations—can significantly increase uptake. The policy makers should also keep in mind that vaccine hesitance in one disease can affect all vaccines. These strategies collectively align with the WHO’s 3Cs model to combat vaccine hesitancy effectively.

Our results demonstrate that stance analysis using LLMs provides a more nuanced understanding than traditional sentiment analysis. Unlike traditional sentiment analysis, which captures general emotional tones, LLMs can identify specific stances such as supportive, opposing, or neutral positions, allowing for a deeper comprehension of public attitudes and decision-making processes. Moreover, the successful application of LLMs highlights their potential as powerful tools for social media analysis, providing deep insights into public health discourse by capturing more complex logic, such as causality and context-specific nuances. These results not only validate the effectiveness of our methodology but also provide strong empirical support for using LLMs for similar tasks in the future.

In future work, we plan to explore why LLMs outperform traditional models by focusing on factors such as the model size and external information. We are also considering building an LLM to identify misinformation regarding HPV vaccines with higher accuracy. We also plan to collect social media data about other vaccines such as COVID-19 to make more solid causality analysis toward the relationship among attitudes toward vaccines. These efforts have refined our understanding of LLMs and improved public health strategies.

### Limitations

Our study has several limitations, including the fact that social media users may not represent the entire population, which limits the generalizability of our findings. We used only X (Twitter) data in this research, which may also affect the representativeness. In addition, these models may introduce classification errors, particularly when addressing nuanced or ambiguous stances and logic. Other factors including but not limited to the biased training data, outdated information, domain adaptation issues, and hallucinations of the LLMs can also lead to inaccuracy of the statistical results. In the logic analysis of relationship between attitudes toward HPV and COVID-19 vaccine, lacking data related to COVID-19 vaccine, we are not able to further prove the causality with methods such as Granger causality tests or structural equation modeling. Furthermore, the keywords used to collect data may have led to the omission of relevant tweets or the addition of irrelevant tweets, potentially affecting the comprehensiveness of our analysis.

### Comparison With Prior Work

Our work stands out from prior studies because of the scale and depth of the dataset, which includes 10 years of tweets in Japanese, providing a comprehensive view of public attitudes over time. We applied both traditional NLP methods and cutting-edge LLMs, which allowed us to analyze nuanced shifts in public sentiment and stance with greater precision. Unlike traditional surveys that provide snapshots of attitudes, our approach captures real-time changes in public discourse. Furthermore, compared with studies relying on Google search trends, our analysis offers more fine-grained insights into specific stances and topics, highlighting not just information-seeking behavior but also the underlying attitudes that drive vaccine hesitancy or advocacy.

### Conclusions

In this study, we provided a decade long examination of public discourse on HPV vaccination in Japan by analyzing social media content with traditional NLP models and LLMs. We also contextualized the complex factors influencing stances toward HPV vaccines under the WHO’s 3Cs model. Public confidence fluctuated significantly in response to government actions and media coverage, indicating the sensitivity of trust in policy decisions, whereas complacency was affected by perceived risks and proactive advocacy. Convenience is crucial for improving vaccine accessibility and for shaping the public’s willingness to be vaccinated. Moreover, we provided some evidence showing that confidence in one vaccine, such as HPV, may influence confidence in others, such as COVID-19, highlighting interconnected public health narratives. Addressing misinformation, enhancing communication, and improving accessibility remain the key strategies for building trust and reducing hesitancy. Future public health strategies can benefit from these insights by designing effective interventions to boost vaccine confidence and uptake.

## Appendix

Multimedia Appendix 1 Data of vaccine uptake and supply from 2013 to 2022.

Multimedia Appendix 2 Results of latent Dirichlet allocation.

Multimedia Appendix 3 All Twitter IDs used in this research.

Multimedia Appendix 4 Comparison of weighted F1-scores among all the models on 300 test data. Bars show the average and SD on 3 test datasets selected by different random seeds. The numbers above the bars show the average of weighted F1-scores.

Multimedia Appendix 5 Details of model selection for stance analysis.

Multimedia Appendix 6 Selection of ratio of training data of difference stances.

Multimedia Appendix 7 Comparison of weighted F1-scores among models trained with different ratios of stance categories (advocate, opposite, and unknown) in the training data. Bars show the average and SD on 3 test datasets selected by different random seeds. The numbers above the bars show the average of weighted F1-scores.

Multimedia Appendix 8 Performance of the final 5 models.

Multimedia Appendix 9 The record of chat with ChatGPT for manuscript correction and refining.

## Abbreviations

BERT: Bidirectional Encoder Representations from Transformers
DL: deep learning
HPV: human papillomavirus
LDA: latent Dirichlet allocation
LLM: large language model
MHLW: Japanese Ministry of Health, Labour and Welfare
NLP: natural language processing
PELT: pruned exact linear time
WHO: World Health Organization
