# Supplementary material for: Understanding Human Papillomavirus Vaccination Hesitancy in Japan Using Social Media: Content Analysis
Source: J Med Internet Res. 2025 Feb 11;27:e68881. doi: 10.2196/68881 (PMC11862774; doi:10.2196/68881)
Supplement: Multimedia Appendix 1 [file jmir_v27i1e68881_app1.docx]

# Vaccine supply

We collected all raw data possible from the meeting record of the Health Sciences Council, MHLW. The period of data can be overlapping.

| Time period | 2013.4~2013.6 | 2013.4~2013.7 | 2013.8~2013.9 | 2013.10~2014.3 | 2014.4~2014.6 | 2014.4~2015.6 |
| --- | --- | --- | --- | --- | --- | --- |
| Total dose | 240623 | 250302 | 13531 | 30224 | 3534 | 54275 |
| Time period | 2015.4~2015.6 | 2015.7~2016.2 | 2016.3~2016.4 | 2016.4~2016.6 | 2016.5~2016.8 | 2016.9~2016.11 |
| Total dose | 3242 | 8577 | 2439 | 2776 | 3615 | 2775 |
| Time period | 2016.12~2017.4 | 2017.4~2017.6 | 2017.5~2017.8 | 2017.9~2017.12 | 2018.1~2018.4 | 2018.5~2018.8 |
| Total dose | 4389 | 2460 | 3828 | 5319 | 7425 | 9288 |
| Time period | 2018.9~2018.12 | 2019.1~2019.4 | 2019.5~2019.8 | 2019.9~2019.12 | 2020.1~2020.4 | 2020.5~2020.9 |
| Total dose | 11489 | 13731 | 16180 | 23140 | 28462 | 56742 |
| Time period | 2020.10~2020.12 | 2021.1~2021.3 | 2021.4~2021.6 | 2021.7~2021.9 | 2021.10~2021.12 | 2022.1~2022.4 |
| Total dose | 78830 | 100759 | 109105 | 168063 | 176194 | 265064 |
| Time period | 2022.5 | 2022.6 | 2022.7 | 2022.8 | 2022.9 | 2022.10~2022.12 |
| Total dose | 72324 | 121807 | 140073 | 193107 | 159885 | 394368 |

## Estimation of total supply in 2013:

The precise data between 2013.10 and 2013.12 is missing. Calculated by the assumption that the supply in each month between 2013.10~2014.3 are equal:

Ñ(2013) = N(2013.4~2013.7) + N(2013.8~2013.9) +N(2013.10~2014.3) / 6 * 3

= 250302+13531+ 30224 / 2

= 278945

## Estimation of total supply in 2014:

The precise data between 2014.1 and 2014.3 is missing. Calculated by the assumption that the supply in each month between 2013.10~2014.3 are equal.

The precise data between 2014.6 and 2014.12 is missing. Calculated by the assumption that the supply in each month between 2014.7~2015.3 are equal.

Ñ(2014) = N(2013.10~2014.3) / 6 * 3 + N(2014.4~2014.6) + [N(2014.4~2015.6) - N(2014.4~2014.6) - N(2015.4~2015.6)] / 9 * 6

= 30224 / 2 + 3534 + (54275 - 3534 - 3242) / 3 * 2

= 50312

## Estimation of total supply in 2015:

The precise data between 2015.1 and 2015.3 is missing. Calculated by the assumption that the supply in each month between 2015.7~2015.3 are equal.

The precise data between 2015.7 and 2015.12 is missing. Calculated by the assumption that the supply in each month between 2015.7~2016.2 are equal.

Ñ(2015) = (N(2014.4~2015.6) - N(2014.4~2014.6) - N(2015.4~2015.6)) / 9 * 3 + N(2015.4~2015.6) + N(2015.7~2016.2) / 8 * 6

= (54275 - 3534 - 3242) / 3 + 3242 + 8577 / 4 * 3

= 25507.75

## Estimation of total supply in 2016:

The precise data between 2016.1 and 2016.2 is missing. Calculated by the assumption that the supply in each month between 2015.7~2016.2 are equal.

The precise data for 2016.12 is missing. Calculated by the assumption that the supply in each month between 2016.12~2017.4 are equal.

Ñ(2016) = N(2015.7~2016.2) / 8 * 2 + N(2016.3~2016.4) + N(2016.5~2016.8) + N(2016.9~2016.11) + N(2016.12~2017.4) / 5

= 8577 / 4 + 2439 + 3615 + 2775 + 4389 / 5

= 11851.05

## Estimation of total supply in 2017:

The precise data between 2017.1 and 2017.3 is missing. Calculated by the assumption that the supply in each month between 2016.12~2017.4 are equal.

Ñ(2016) = N(2016.12~2017.4) / 5 * 4 + N(2017.5~2017.8) + N(2017.9~2017.12)

= 4389 / 5 * 4 + 3828 + 5319

= 12658.2

# Vaccine uptake

Table 2. the yearly record of accepted vaccine doses.

| Year | First dose | Second dose | Third dose |
| --- | --- | --- | --- |
| 2013 | 98656 | 66568 | 87233 |
| 2014 | 3895 | 4172 | 6238 |
| 2015 | 2711 | 2669 | 2805 |
| 2016 | 1834 | 1805 | 1782 |
| 2017 | 3347 | 2666 | 1847 |
| 2018 | 6810 | 5746 | 4184 |
| 2019 | 17297 | 13571 | 9701 |
| 2020 | 83735 | 61266 | 37556 |
| 2021 | 198474 | 182463 | 139014 |
| 2022 | 540681 | 476322 | 336762 |
