# Supplementary material for: Understanding Human Papillomavirus Vaccination Hesitancy in Japan Using Social Media: Content Analysis
Source: J Med Internet Res. 2025 Feb 11;27:e68881. doi: 10.2196/68881 (PMC11862774; doi:10.2196/68881)
Supplement: Multimedia Appendix 2 [file jmir_v27i1e68881_app2.docx]

Table S1. COVID-19 vaccine-related key events

| Date | Event (negative) | Date | Event (positive) |
| --- | --- | --- | --- |
| 2020-08-24 | Drug victim groups request careful verification for vaccine approval | 2020-11-10 | Pfizer announces interim results: vaccine 'over 90% effective |
| 2020-09-09 | AstraZeneca temporarily halts clinical trials of COVID-19 vaccine | 2020-12-11 | US FDA grants emergency use authorization for Pfizer's vaccine |
| 2020-12-16 | Vaccine recipients in the US show symptoms of possible allergic reactions | 2021-02-14 | Japan's Ministry of Health approves Pfizer vaccine, first official approval in the country |
| 2020-12-19 | Six people in the US experience severe allergic symptoms after vaccination | 2021-04-12 | Vaccination for elderly begins |
| 2021-01-06 | 21 out of about 1.9 million people show severe allergic reactions to Pfizer vaccine in the US | 2021-05-24 | Mass vaccination sites open in Tokyo and Osaka |
| 2021-02-03 | Vaccine side effect rate is 0.24% in Israel, where about 30% of population is vaccinated | 2021-08-24 | US FDA fully approves Pfizer vaccine |
| 2021-03-12 | Reports of blood clots associated with AstraZeneca vaccine | 2021-08-28 | Two deaths after vaccination postponed due to contamination issue, causal link unclear |
| 2021-06-24 | CDC: Myocarditis 'possibly related to COVID-19 vaccine’ | 2021-09-12 | Kyoto University analysis shows COVID-19 vaccine effectiveness between 85% to over 90% |
| 2021-07-02 | Large-scale national survey: 11% 'don't want to be vaccinated', mostly younger generation | 2021-10-22 | Pfizer: Third dose of vaccine '95.6% effective' according to clinical trial results |
|  |  | 2021-11-11 | COVID-19 vaccine 87% effective, 'possibly highly effective against Delta variant' |

## LDA on all HPV vaccine-related tweets


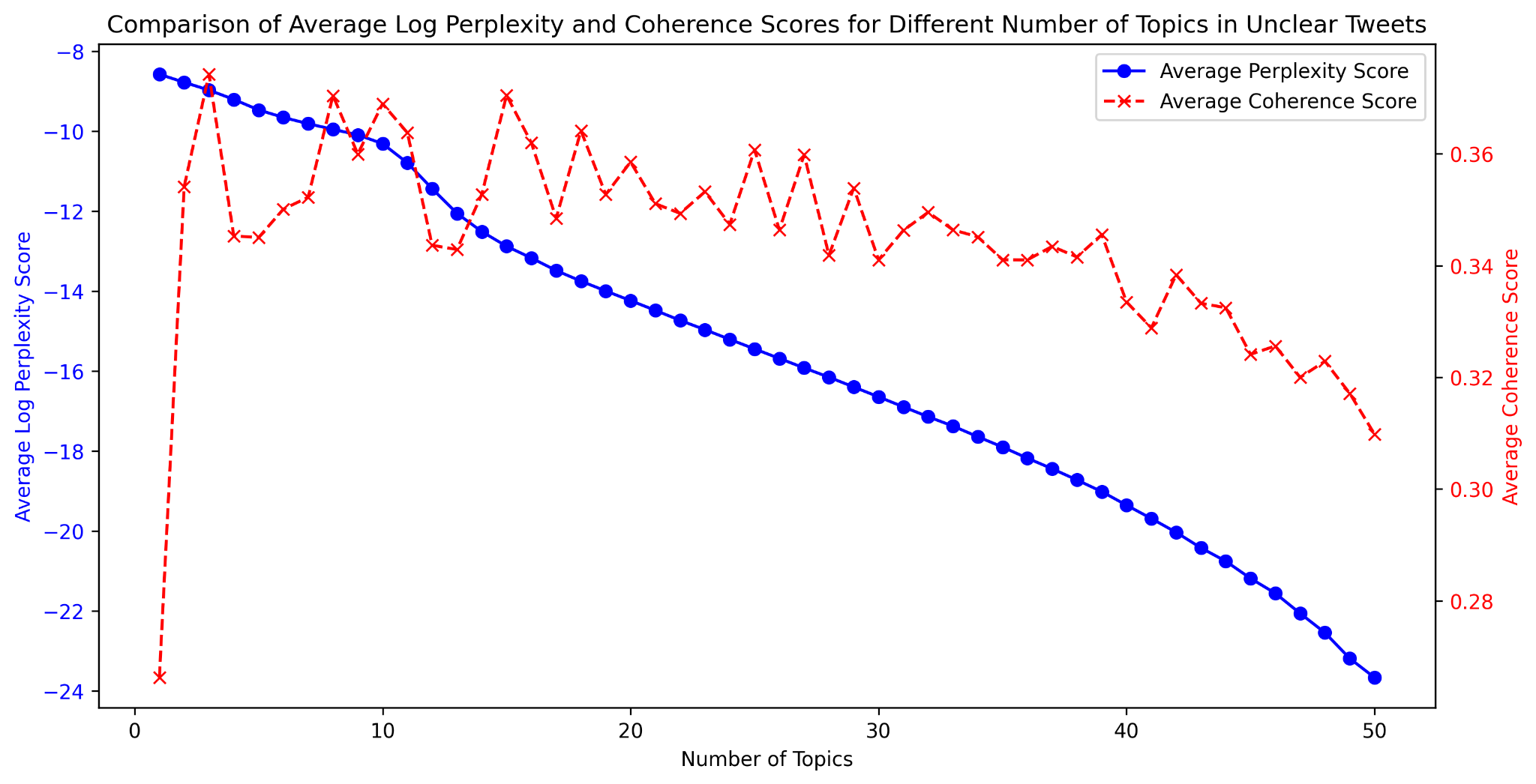


Figure S1. Average Perplexity and Coherence scores for LDA topic selection, unknown stance.


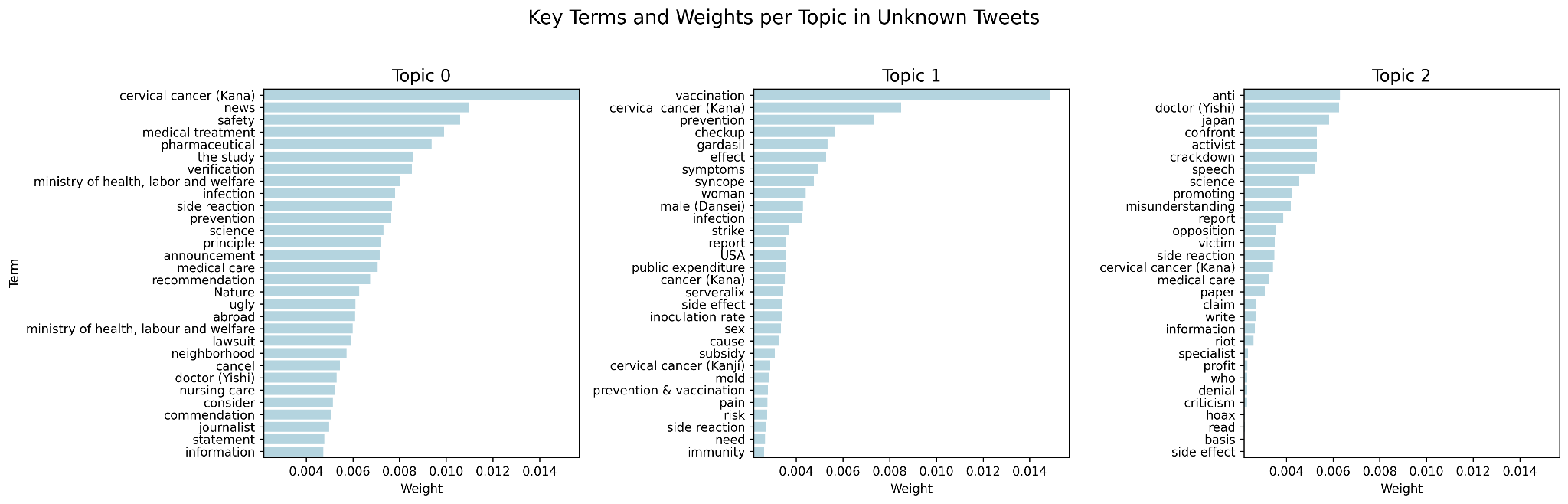


Figure S2. Top 30 words of each topic of unknown stance.


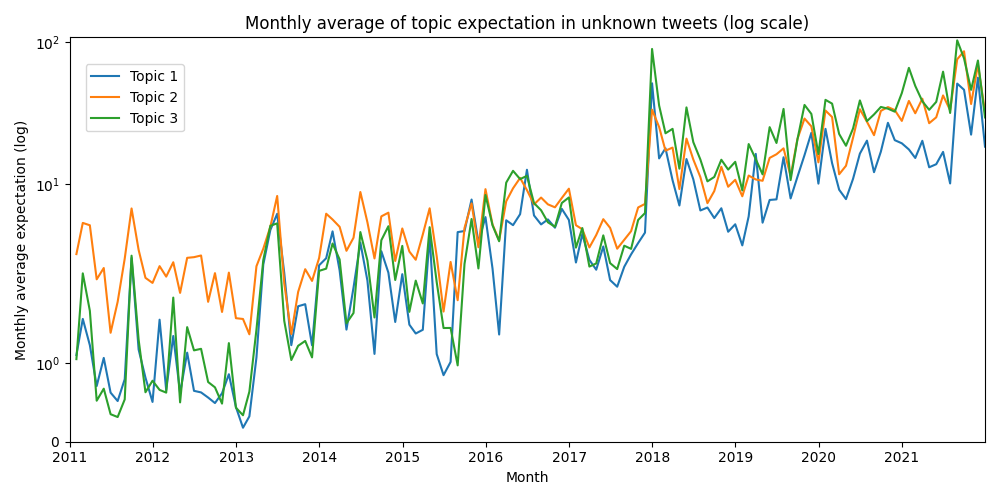


Figure S3. Monthly average of topic expectation in unknown tweets. The number is in log scale.


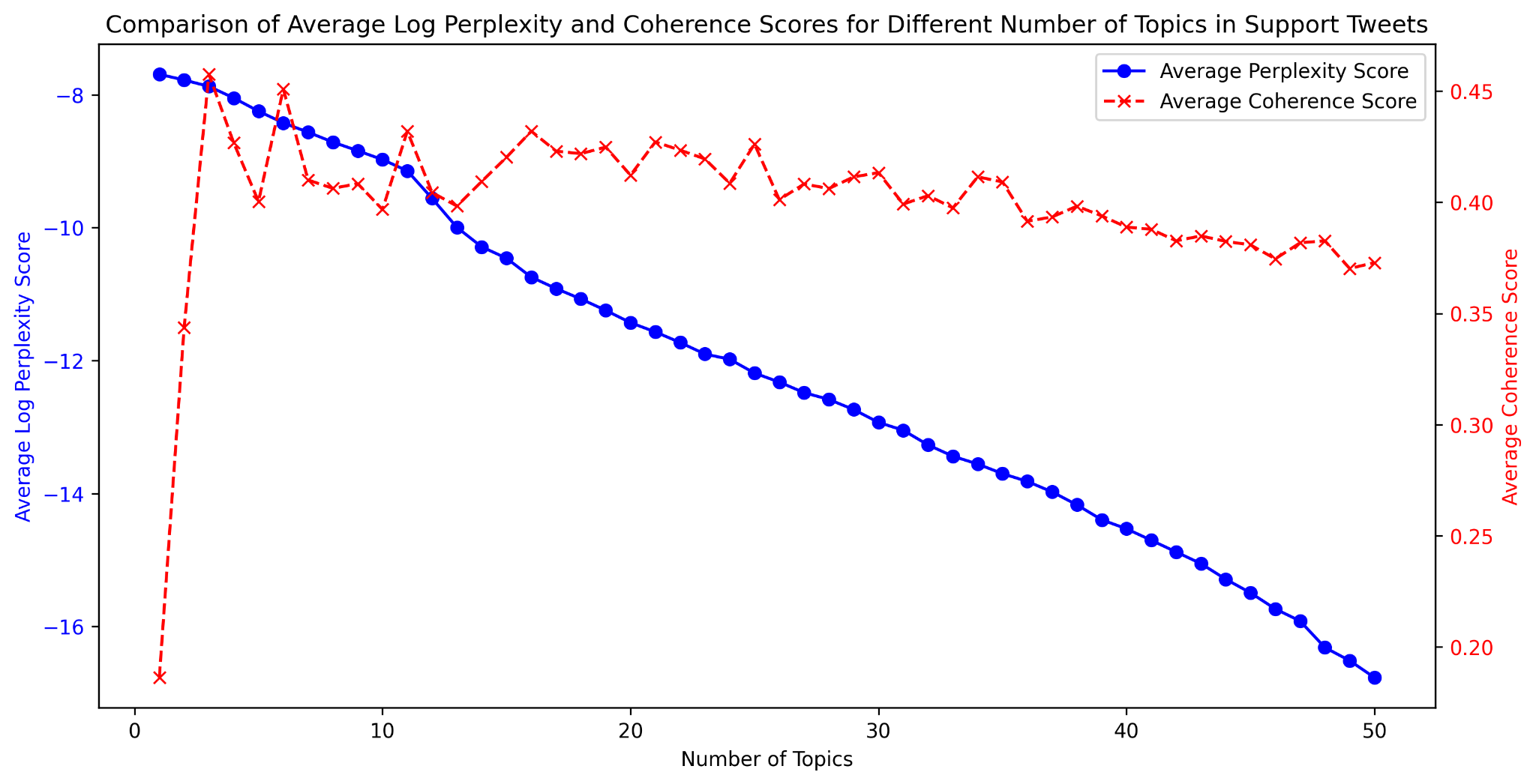


Figure S4. Average Perplexity and Coherence scores for LDA topic selection, advocate stance.


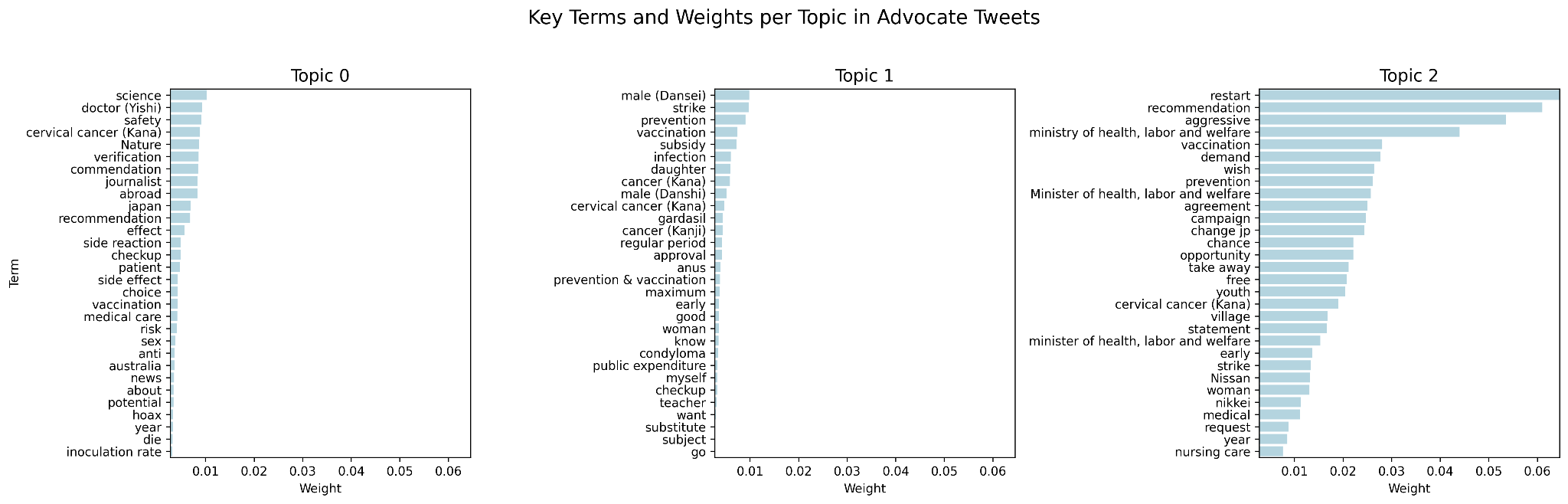


Figure S5. Top 30 words of each topic of advocate stance.


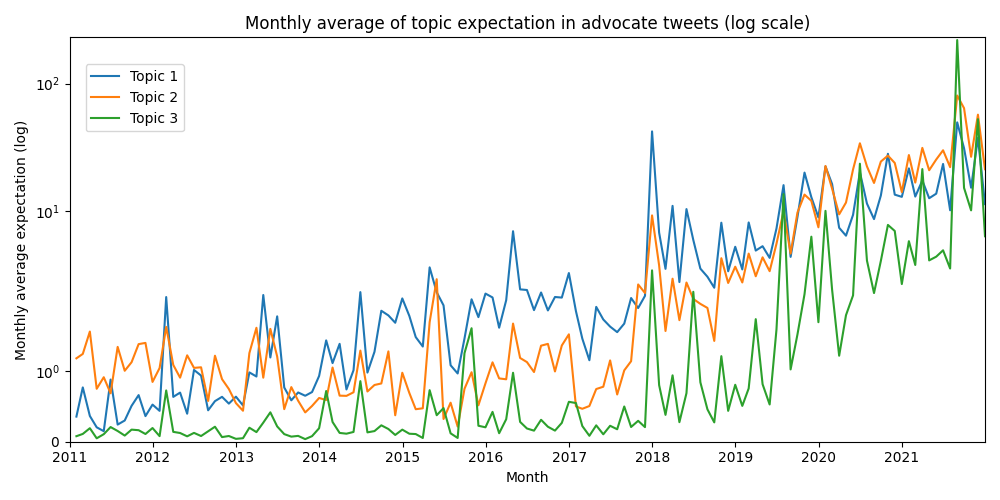


Figure S6. Monthly average of topic expectation in advocate tweets. The number is in log scale.

# Opposition:


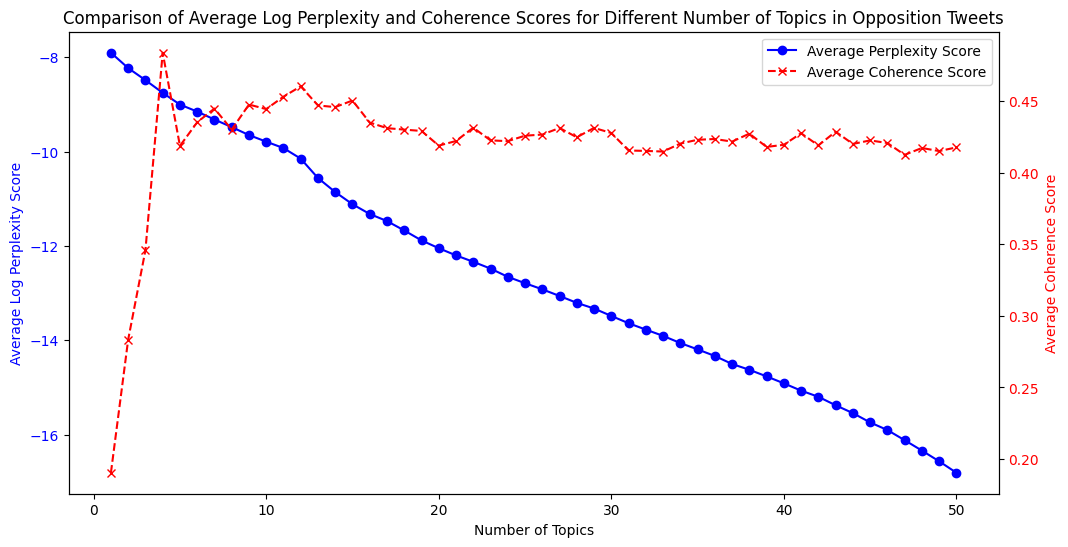


Figure S7. Average Perplexity and Coherence scores for LDA topic selection, opposed stance.


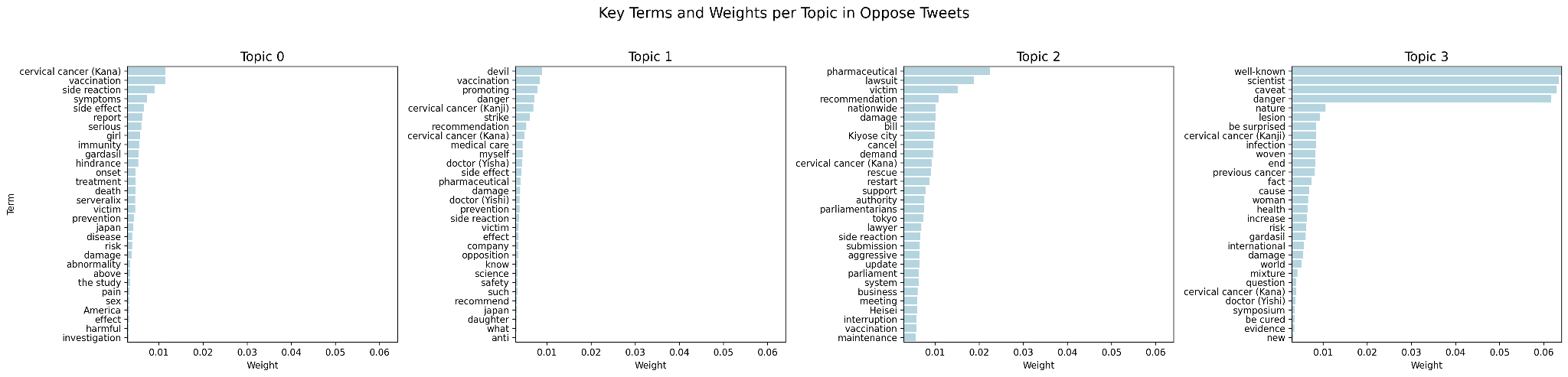


Figure S8. Top 30 words of each topic of opposed stance.
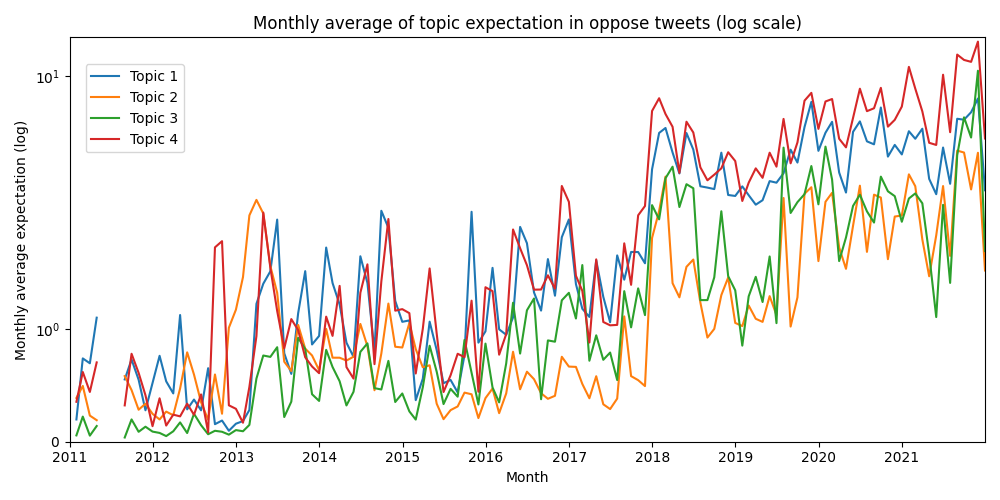


Figure S9. Monthly average of topic expectation in opposed tweets. The number is in log scale.

### LDA topics:

Unknown:

1. “HPV Vaccine Safety and Governmental Oversight” - This topic centers around the safety and regulation of the HPV vaccine in the context of cervical cancer. It includes discussions related to government bodies like the Ministry of Health, Labour and Welfare, safety concerns, adverse reactions, and the role of the medical system. There are also mentions of vaccine-related legal cases, the suspension of vaccination programs, and the importance of scientific research in assessing vaccine safety. This topic highlights public health measures and controversies surrounding the HPV vaccine, both domestically and internationally.
2. “HPV Vaccine Efficacy and Public Health Initiatives” - This topic revolves around the administration of the HPV vaccine and its role in cancer prevention , particularly for cervical cancer and other related conditions. It discusses the effectiveness of vaccines like Gardasil and Cervarix, as well as reported symptoms and adverse effects such as fainting following vaccination. There is a focus on both women and men and the importance of public funding for vaccination programs and vaccination rates. The topic also touches on infection risks, immune response, and the need for preventive healthcare measures like regular screenings.
3. “Opposition, Misinformation, and Activism Surrounding HPV Vaccination” - This topic covers the opposition to HPV vaccination and related activism. It includes discussions about the suppression of dissent, issues related to freedom of speech, and the spread of misinformation. It highlights the involvement of both physicians and activists, along with debates over scientific evidence and the media's role in reporting on the issue. There are mentions of victims, adverse reactions, and critiques of the medical system, particularly in Japan. This topic captures the contentious dialogue between supporters and critics of the vaccine, as well as the broader societal and political implications of the debate.

Advocate:

1. “Scientific and media discourse on HPV vaccine safety” - This topic revolves around scientific discussions and media reports concerning the safety and efficacy of the HPV vaccine. It includes references to scientists, medical professionals, and the safety of the vaccine in preventing cervical cancer. Terms like "Nature", a prominent scientific journal, suggest a focus on studies and verification of vaccine outcomes. The involvement of journalists and international perspectives indicates that this topic captures both the scientific and public discussion about the vaccine, including its promotion and potential side effects.
2. “HPV vaccine effectiveness and broader public health measures” - This topic focuses on the administration of the HPV vaccine to both males and females, addressing preventive measures and public health initiatives. The discussion includes references to male-specific cancers, and the inclusion of boys in vaccination programs. It touches on the importance of regular vaccination and cancer prevention, not just for cervical cancer, but other forms of HPV-related cancers. The topic reflects public outreach efforts to include men in vaccination campaigns, as well as public understanding and awareness of the vaccine’s broader health benefits.
3. “Policy and advocacy for HPV vaccination promotion” - This topic deals with policy initiatives and advocacy for the resumption of HPV vaccination recommendations in Japan, led by the Ministry of Health, Labour and Welfare. It includes efforts to promote the vaccine and the involvement of campaigns to raise awareness. Words like "petition" (changejp) and "free" highlight advocacy efforts to make the vaccine more accessible, particularly for young people. This topic captures the ongoing debate and the role of government in ensuring access to the vaccine and promoting public health.

Opposite:

1. “HPV vaccine adverse effects” - This topic seems to revolve around the HPV vaccine, including discussions about cervical cancer, side effects, and the immune response. It also mentions specific vaccines like Gardasil and Cervarix, alongside issues such as serious adverse effects, potential risk , and cases of reported damage or injury. This suggests a focus on the medical aspects of HPV vaccination, its reported effectiveness, and concerns about adverse reactions, especially among young females.
2. “Skepticism and opposition to vaccination” - This topic captures the concerns and resistance surrounding HPV vaccinations. Words like "danger", "devil", "self", and "recommendation" point to a more skeptical or critical perspective on the vaccine. There is a strong emphasis on opposition to vaccination, distrust in the medical community, and claims of pharmaceutical involvement . The presence of words like "danger" and "devil" may indicate strong emotional or ideological opposition, linking to fears about safety, scientific skepticism, and distrust of institutions promoting vaccination.
3. “Legal and social responses to vaccine-related harm” - This topic involves discussions around legal and social actions, including lawsuits related to vaccine injuries and the legal framework for addressing vaccine-related harm. Words like "lawsuits", "victims", "national", and "compensation" highlight concerns about injury claims and the demand for government action. The topic references efforts to halt or restart vaccination programs, as well as calls for policy changes in Japan. There's also an emphasis on legal groups and local government action.
4. “Scientific warnings and public health risks” - This topic focuses on warnings from scientists about the potential risks associated with vaccination, with a clear focus on public health concerns. Words like "danger", "warning" , "infection", and "risk" suggest that the discourse is centered around the scientific debate on the safety and health impact of the HPV vaccine. The references to "international" and "global" contexts may indicate broader concerns about vaccine-related health risks at an international level. This topic reflects a dialogue on the scientific community's role in informing the public about potential risks and benefits.

## LDA on tweets with “COVID-19” keywords


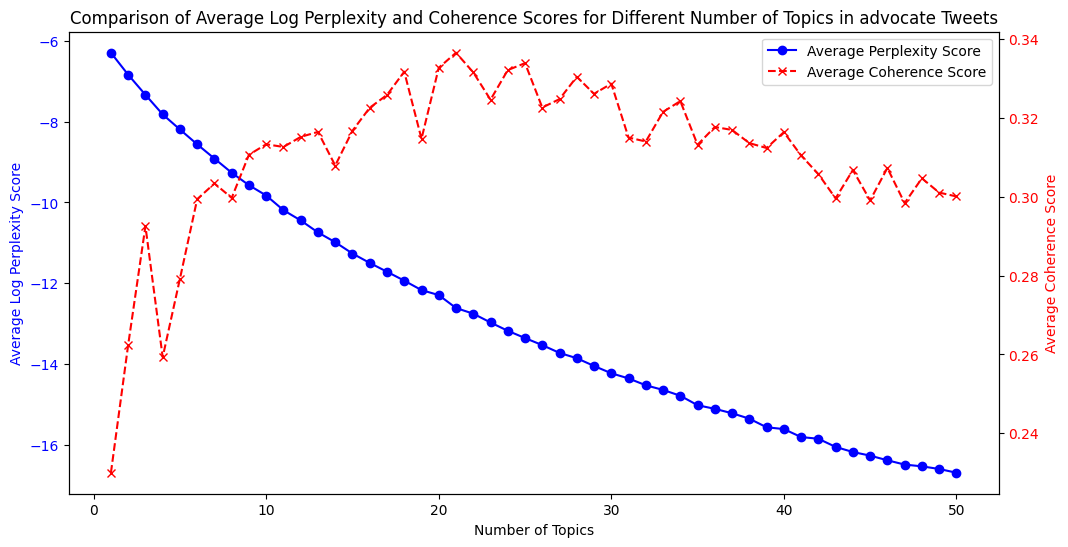


Figure S10. Average Perplexity and Coherence scores for LDA topic selection, advocate stance.


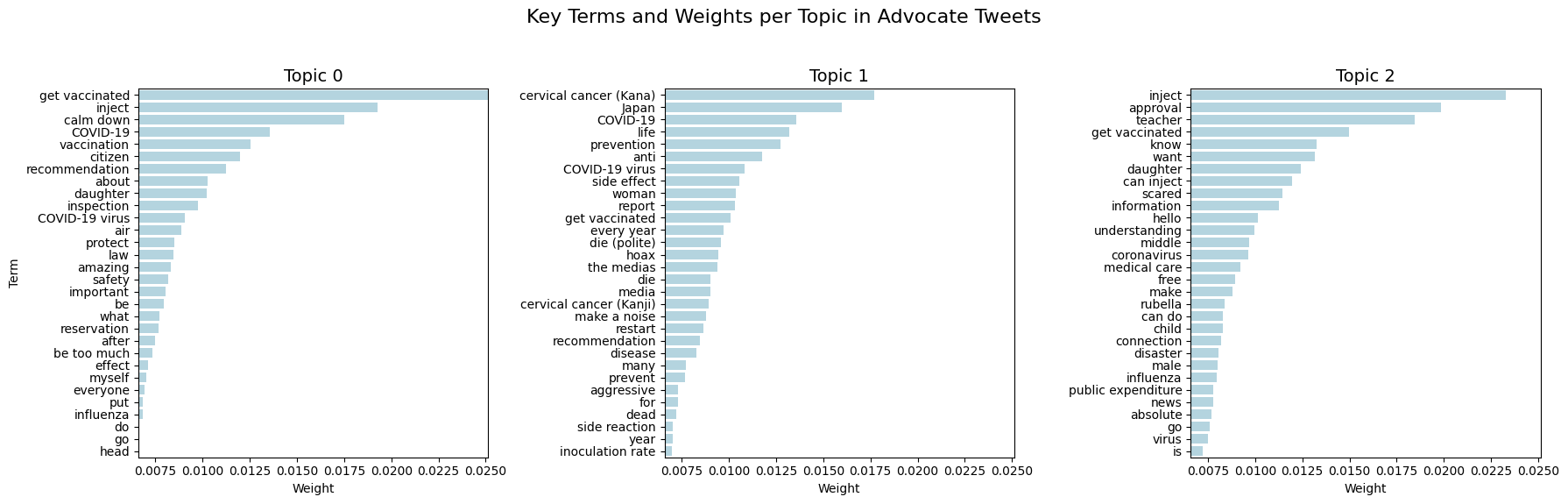


Figure S11. Top 30 words of each topic of advocate stance.


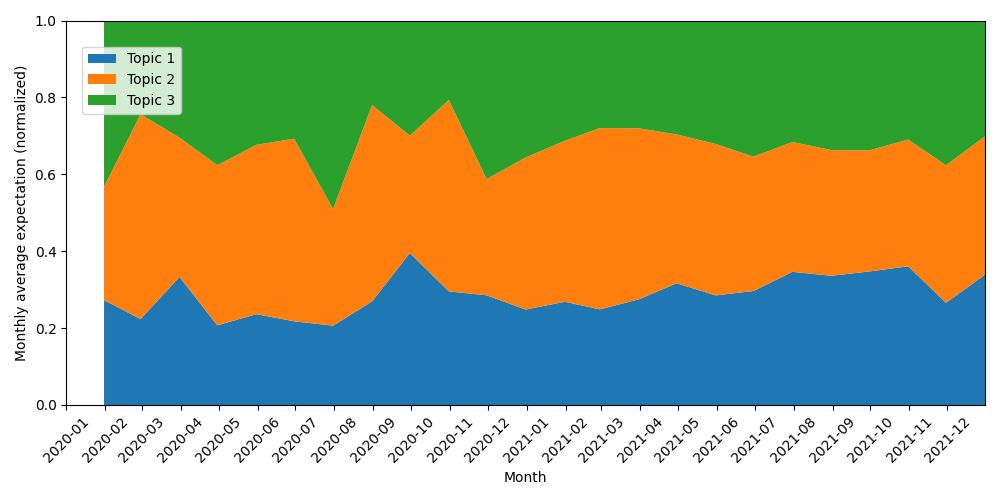


Figure S12. Weekly change of ratio of different topics in tweets of advocate stance and containing “COVID-19” keywords from 2020 to 2021.


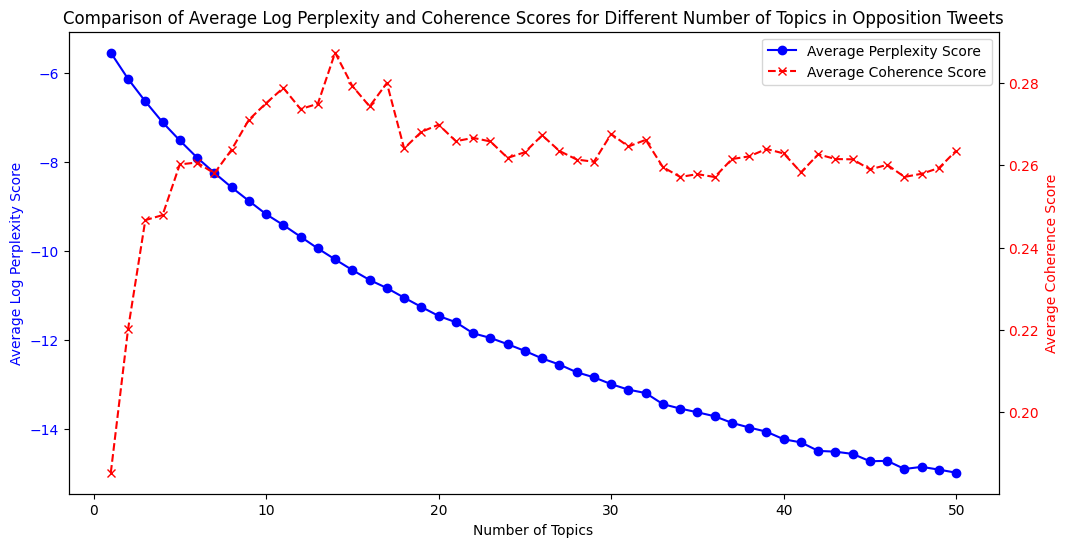


Figure S13. Average Perplexity and Coherence scores for LDA topic selection, opposed stance.


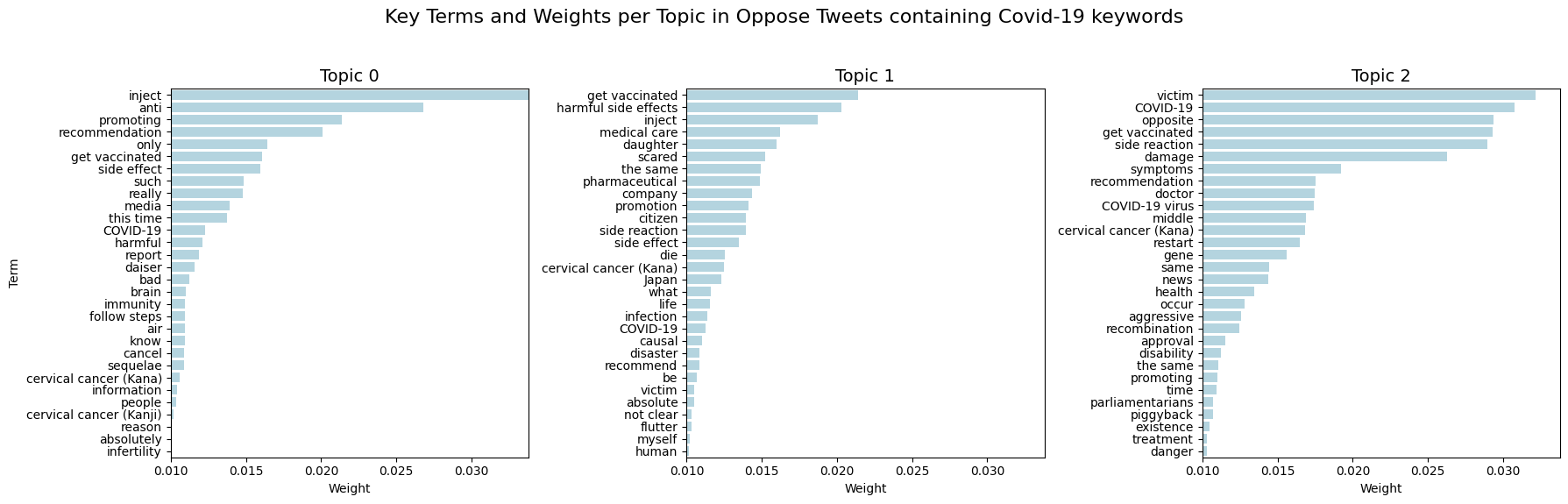


Figure S14. Top 30 words of each topic of opposed stance.

#
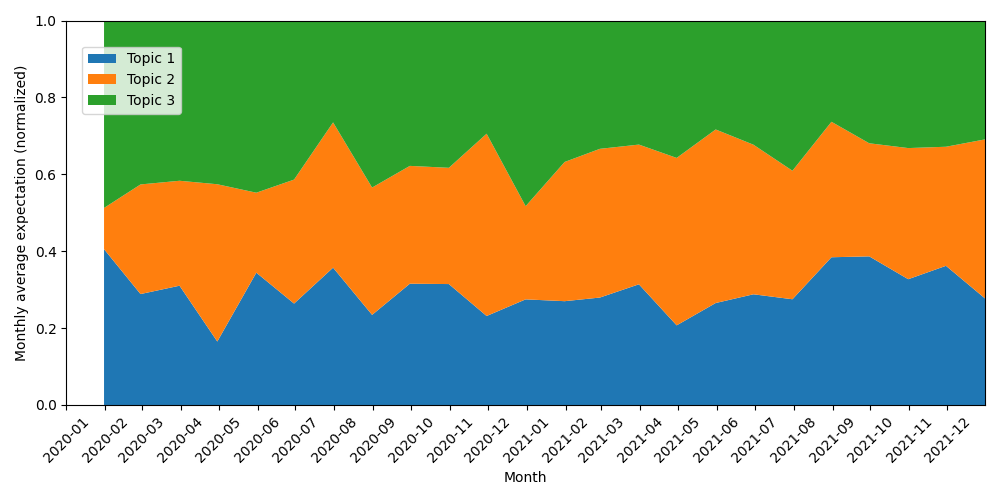


Figure S15. Weekly change of ratio of different topics in tweets of opposed stance and containing “COVID-19” keywords from 2020 to 2021.

### LDA Topics on tweets containing “COVID-19” keywords:

Advocate:

- "Advocating the importance and safety of vaccination" - This theme emphasizes the importance and positivity of vaccination. Keywords such as “vaccination”, "vaccination”, “calm/assured”,, “preventive vaccination”, “safety”, and “effectiveness” indicate trust in the efficacy and safety of vaccines. Mentions of “daughter” and “protect” reflect a desire to protect family health through vaccination.
- "Promoting cervical Cancer prevention and countering misinformation" - This theme focuses on the importance of cervical cancer prevention. Keywords include “cervical cancer”, “prevention”, “to die”, “to prevent”, and “vaccination rate”, emphasizing the role of vaccines in preventing diseases and saving lives. Mentions of “rumors” and “media” indicate a resistance to misinformation, along with a desire for accurate media reporting on vaccines.
- "Enhancing public understanding through education and medical support" - This theme centers on strengthening vaccine knowledge dissemination and alleviating public fear. Keywords such as “vaccination”, “approval”, “doctor”, “know”, “information”, and “understanding” reflect efforts to enhance public understanding and trust in vaccines through doctors and accurate information. Mentions of “free” and “public funding” suggest government initiatives to provide free vaccinations, along with calls to encourage men to get vaccinated. In summary, the themes from opponents primarily revolve around questioning vaccine safety, distrust towards pharmaceutical companies, and criticism of government and media roles in vaccine promotion. Supporters' themes emphasize the importance of vaccines in disease prevention, protecting lives, urging public trust in science, resisting misinformation, and enhancing vaccine education efforts.

Opposite:

- "Concerns about vaccine promotion and potential side effects" - This theme focuses on opposition to vaccine promotion and recommendations, primarily addressing concerns about vaccine side effects and potential harms. Keywords such as “vaccination”, “oppose”, “promotion”, “side effects”, “harmful”, and “infertility” indicate worries about vaccine safety. Additionally, mentions of “media” and “reporting” suggest dissatisfaction with media hype surrounding vaccines, as well as concerns about serious health issues that vaccines might cause, such as “brain” and “aftereffects”.
- "Distrust of pharmaceutical companies and fear of severe adverse reactions" - This theme expresses distrust towards pharmaceutical companies and medical institutions. Keywords include “drug harm”, “pharmaceutical”, “company”, “advertising”, and “adverse reactions”, along with “die”, reflecting fears of severe side effects or even death from vaccines. The mention of “daughter” indicates particular concern for female family members receiving vaccinations.
- "Vaccine victims, genetic engineering worries, and criticism of government policies" - This theme discusses the voices of vaccine victims and opposition to vaccination. Keywords like “victim”, “oppose”, “adverse reactions”, “harm”, and “disability” emphasize doubts about vaccine safety. Mentions of “genes” and “recombinant” may involve concerns regarding vaccine technology, as well as questioning the roles of government and political figures in vaccine promotion. Supporters' Themes:
