# Supplementary figures and images for: Understanding Human Papillomavirus Vaccination Hesitancy in Japan Using Social Media: Content Analysis
Source: J Med Internet Res. 2025 Feb 11;27:e68881. doi: 10.2196/68881 (PMC11862774; doi:10.2196/68881)

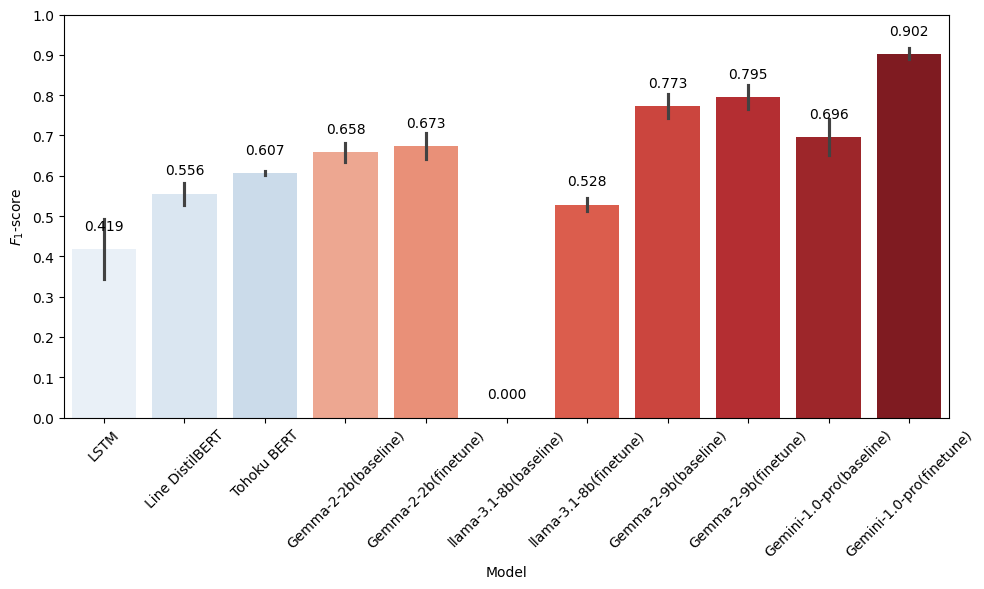

Supplement: Multimedia Appendix 4 [file jmir_v27i1e68881_app4.png]

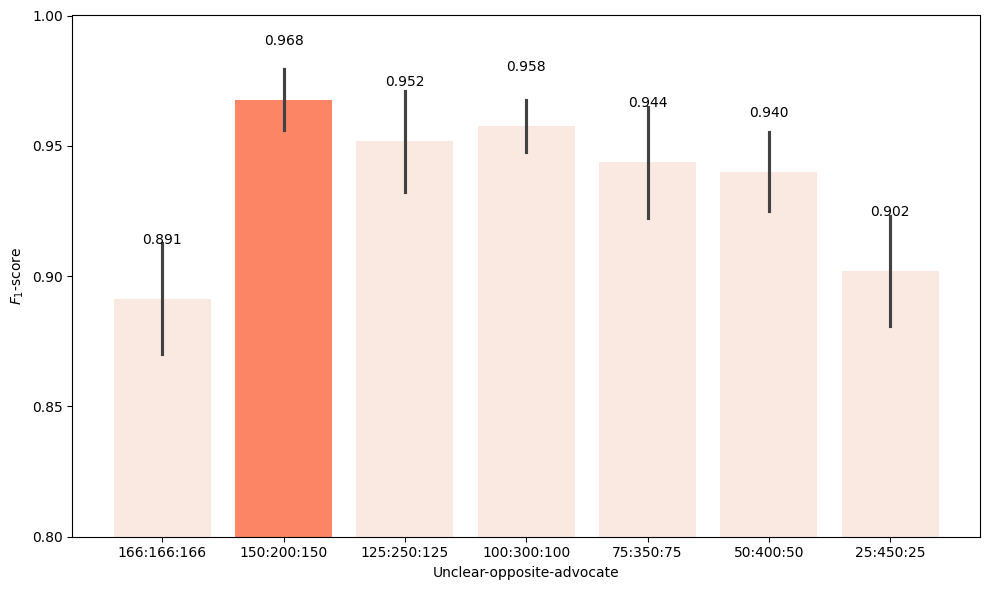

Supplement: Multimedia Appendix 7 [file jmir_v27i1e68881_app7.png]
