# Supplementary material for: Understanding Human Papillomavirus Vaccination Hesitancy in Japan Using Social Media: Content Analysis
Source: J Med Internet Res. 2025 Feb 11;27:e68881. doi: 10.2196/68881 (PMC11862774; doi:10.2196/68881)
Supplement: Multimedia Appendix 5 [file jmir_v27i1e68881_app5.docx]

## Results on traditional models

All models were finetuned three times, with 100 test data randomly selected from each stance. The remaining data within each category was then partitioned into training and validation sets at a ratio of 4:1.

| **Table S1. results on 1st round, LSTM, random seed=1** | | | | |
| --- | --- | --- | --- | --- |
|  | Precision | Recall | F1-score | Support |
| Unclear | 0.399 | 0.930 | 0.559 | 100 |
| Opposite | 0.875 | 0.070 | 0.130 | 100 |
| Advocate | 0.695 | 0.410 | 0.516 | 100 |
|  |  |  |  |  |
| Accuracy | - | - | 0.470 | 300 |
| Macro avg | 0.656 | 0.470 | 0.401 | 300 |
| Weighted avg | 0.656 | 0.470 | 0.401 | 300 |

| **Table S2. results on 2nd round, LSTM, random seed=2** | | | | |
| --- | --- | --- | --- | --- |
|  | Precision | Recall | F1-score | Support |
| Unclear | 0.438 | 0.910 | 0.591 | 100 |
| Opposite | 0.895 | 0.170 | 0.286 | 100 |
| Advocate | 0.740 | 0.540 | 0.624 | 100 |
|  |  |  |  |  |
| Accuracy | - | - | 0.540 | 300 |
| Macro avg | 0.691 | 0.540 | 0.500 | 300 |
| Weighted avg | 0.691 | 0.540 | 0.500 | 300 |

| **Table S3. results on 3rd round, LSTM, random seed=3** | | | | |
| --- | --- | --- | --- | --- |
|  | Precision | Recall | F1-score | Support |
| Unclear | 0.379 | 0.920 | 0.536 | 100 |
| Opposite | 0.667 | 0.080 | 0.143 | 100 |
| Advocate | 0.622 | 0.280 | 0.386 | 100 |
|  |  |  |  |  |
| Accuracy | - | - | 0.427 | 300 |
| Macro avg | 0.556 | 0.427 | 0.355 | 300 |
| Weighted avg | 0.556 | 0.427 | 0.355 | 300 |

| **Table S4. results on 1st round, Distill bert, random seed=1** | | | | |
| --- | --- | --- | --- | --- |
|  | Precision | Recall | F1-score | Support |
| Unclear | 0.471 | 0.730 | 0.573 | 100 |
| Opposite | 0.571 | 0.280 | 0.376 | 100 |
| Advocate | 0.635 | 0.610 | 0.622 | 100 |
|  |  |  |  |  |
| Accuracy | - | - | 0.540 | 300 |
| Macro avg | 0.559 | 0.540 | 0.524 | 300 |
| Weighted avg | 0.559 | 0.540 | 0.524 | 300 |

| **Table S5. results on 2nd round, Distill bert, random seed=2** | | | | |
| --- | --- | --- | --- | --- |
|  | Precision | Recall | F1-score | Support |
| Unclear | 0.514 | 0.730 | 0.603 | 100 |
| Opposite | 0.616 | 0.450 | 0.520 | 100 |
| Advocate | 0.635 | 0.540 | 0.584 | 100 |
|  |  |  |  |  |
| Accuracy | - | - | 0.573 | 300 |
| Macro avg | 0.589 | 0.573 | 0.569 | 300 |
| Weighted avg | 0.589 | 0.573 | 0.569 | 300 |

| **Table S6. results on 3rd round, Distill bert, random seed=3** | | | | |
| --- | --- | --- | --- | --- |
|  | Precision | Recall | F1-score | Support |
| Unclear | 0.483 | 0.730 | 0.582 | 100 |
| Opposite | 0.717 | 0.380 | 0.497 | 100 |
| Advocate | 0.656 | 0.630 | 0.643 | 100 |
|  |  |  |  |  |
| Accuracy | - | - | 0.580 | 300 |
| Macro avg | 0.619 | 0.580 | 0.574 | 300 |
| Weighted avg | 0.619 | 0.580 | 0.574 | 300 |

| **Table S7. results on 1st round, Bert, random seed=1** | | | | |
| --- | --- | --- | --- | --- |
|  | Precision | Recall | F1-score | Support |
| Unclear | 0.516 | 0.650 | 0.575 | 100 |
| Opposite | 0.730 | 0.540 | 0.621 | 100 |
| Advocate | 0.630 | 0.630 | 0.630 | 100 |
|  |  |  |  |  |
| Accuracy | - | - | 0.607 | 300 |
| Macro avg | 0.625 | 0.607 | 0.609 | 300 |
| Weighted avg | 0.625 | 0.607 | 0.609 | 100 |

| **Table S8. results on 2nd round, Bert, random seed=2** | | | | |
| --- | --- | --- | --- | --- |
|  | Precision | Recall | F1-score | Support |
| Unclear | 0.529 | 0.630 | 0.575 | 100 |
| Opposite | 0.587 | 0.610 | 0.598 | 100 |
| Advocate | 0.727 | 0.560 | 0.633 | 100 |
|  |  |  |  |  |
| Accuracy | - | - | 0.600 | 300 |
| Macro avg | 0.614 | 0.600 | 0.602 | 300 |
| Weighted avg | 0.614 | 0.600 | 0.602 | 100 |

| **Table S9. results on 3rd round, Bert, random seed=3** | | | | |
| --- | --- | --- | --- | --- |
|  | Precision | Recall | F1-score | Support |
| Unclear | 0.549 | 0.730 | 0.627 | 100 |
| Opposite | 0.649 | 0.480 | 0.552 | 100 |
| Advocate | 0.677 | 0.630 | 0.653 | 100 |
|  |  |  |  |  |
| Accuracy | - | - | 0.613 | 300 |
| Macro avg | 0.625 | 0.613 | 0.610 | 300 |
| Weighted avg | 0.625 | 0.613 | 0.610 | 100 |

## Results on open source LLMs

All baseline or finetuned models were tested three times, with 100 test data randomly selected from each stance. The remaining data within each category was then partitioned into training and validation sets at a ratio of 4:1 (baseline models were not finetuned).

| **Table S10. results on 1st round, Gemma-2-2b-it (baseline), random seed=1** | | | | |
| --- | --- | --- | --- | --- |
|  | Precision | Recall | F1-score | Support |
| Unclear | 0.611 | 0.580 | 0.595 | 100 |
| Opposite | 0.589 | 0.830 | 0.689 | 100 |
| Advocate | 0.922 | 0.590 | 0.720 | 100 |
|  |  |  |  |  |
| Accuracy | - | - | 0.667 | 300 |
| Macro avg | 0.707 | 0.667 | 0.668 | 300 |
| Weighted avg | 0.707 | 0.667 | 0.668 | 300 |

| **Table S11. results on 2nd round, Gemma-2-2b-it (baseline), random seed=2** | | | | |
| --- | --- | --- | --- | --- |
|  | Precision | Recall | F1-score | Support |
| Unclear | 0.581 | 0.610 | 0.595 | 100 |
| Opposite | 0.597 | 0.860 | 0.705 | 100 |
| Advocate | 0.882 | 0.450 | 0.596 | 100 |
|  |  |  |  |  |
| Accuracy | - | - | 0.640 | 300 |
| Macro avg | 0.687 | 0.640 | 0.632 | 300 |
| Weighted avg | 0.687 | 0.640 | 0.632 | 300 |

| **Table S12. results on 3rd round, Gemma-2-2b-it (baseline), random seed=3** | | | | |
| --- | --- | --- | --- | --- |
|  | Precision | Recall | F1-score | Support |
| Unclear | 0.630 | 0.580 | 0.604 | 100 |
| Opposite | 0.618 | 0.840 | 0.712 | 100 |
| Advocate | 0.847 | 0.640 | 0.632 | 100 |
|  |  |  |  |  |
| Accuracy | - | - | 0.677 | 300 |
| Macro avg | 0.698 | 0.677 | 0.675 | 300 |
| Weighted avg | 0.698 | 0.677 | 0.675 | 300 |

| **Table S13. results on 1st round, Gemma-2-2b-it (finetune), random seed=1** | | | | |
| --- | --- | --- | --- | --- |
|  | Precision | Recall | F1-score | Support |
| Unclear | 0.640 | 0.550 | 0.591 | 100 |
| Opposite | 0.664 | 0.830 | 0.738 | 100 |
| Advocate | 0.820 | 0.730 | 0.772 | 100 |
|  |  |  |  |  |
| Accuracy | - | - | 0.703 | 300 |
| Macro avg | 0.708 | 0.703 | 0.701 | 300 |
| Weighted avg | 0.708 | 0.703 | 0.701 | 300 |

| **Table S14. results on 2nd round, Gemma-2-2b-it (finetune), random seed=2** | | | | |
| --- | --- | --- | --- | --- |
|  | Precision | Recall | F1-score | Support |
| Unclear | 0.729 | 0.350 | 0.473 | 100 |
| Opposite | 0.600 | 0.870 | 0.710 | 100 |
| Advocate | 0.710 | 0.760 | 0.734 | 100 |
|  |  |  |  |  |
| Accuracy | - | - | 0.660 | 300 |
| Macro avg | 0.680 | 0.660 | 0.639 | 300 |
| Weighted avg | 0.680 | 0.660 | 0.639 | 300 |

| **Table S15. results on 3rd round, Gemma-2-2b-it (finetune), random seed=3** | | | | |
| --- | --- | --- | --- | --- |
|  | Precision | Recall | F1-score | Support |
| Unclear | 0.556 | 0.700 | 0.619 | 100 |
| Opposite | 0.688 | 0.750 | 0.718 | 100 |
| Advocate | 0.892 | 0.580 | 0.703 | 100 |
|  |  |  |  |  |
| Accuracy | - | - | 0.677 | 300 |
| Macro avg | 0.712 | 0.677 | 0.680 | 300 |
| Weighted avg | 0.712 | 0.677 | 0.680 | 300 |

| **Table S16. results on 1st round, Gemma-2-9b-it (baseline), random seed=1** | | | | |
| --- | --- | --- | --- | --- |
|  | Precision | Recall | F1-score | Support |
| Unclear | 0.687 | 0.680 | 0.683 | 100 |
| Opposite | 0.756 | 0.900 | 0.822 | 100 |
| Advocate | 0.902 | 0.740 | 0.813 | 100 |
|  |  |  |  |  |
| Accuracy | - | - | 0.773 | 300 |
| Macro avg | 0.782 | 0.773 | 0.773 | 300 |
| Weighted avg | 0.782 | 0.773 | 0.773 | 300 |

| **Table S17. results on 2nd round, Gemma-2-9b-it (baseline), random seed=2** | | | | |
| --- | --- | --- | --- | --- |
|  | Precision | Recall | F1-score | Support |
| Unclear | 0.691 | 0.650 | 0.670 | 100 |
| Opposite | 0.685 | 0.890 | 0.774 | 100 |
| Advocate | 0.908 | 0.690 | 0.784 | 100 |
|  |  |  |  |  |
| Accuracy | - | - | 0.743 | 300 |
| Macro avg | 0.761 | 0.743 | 0.743 | 300 |
| Weighted avg | 0.761 | 0.743 | 0.743 | 300 |

| **Table S18. results on 3rd round, Gemma-2-9b-it (baseline), random seed=3** | | | | |
| --- | --- | --- | --- | --- |
|  | Precision | Recall | F1-score | Support |
| Unclear | 0.731 | 0.790 | 0.760 | 100 |
| Opposite | 0.784 | 0.910 | 0.843 | 100 |
| Advocate | 0.934 | 0.710 | 0.807 | 100 |
|  |  |  |  |  |
| Accuracy | - | - | 0.803 | 300 |
| Macro avg | 0.817 | 0.803 | 0.803 | 300 |
| Weighted avg | 0.817 | 0.803 | 0.803 | 300 |

| **Table S19. results on 1st round, Gemma-2-9b-it (finetune), random seed=1** | | | | |
| --- | --- | --- | --- | --- |
|  | Precision | Recall | F1-score | Support |
| Unclear | 0.802 | 0.650 | 0.718 | 100 |
| Opposite | 0.802 | 0.930 | 0.861 | 100 |
| Advocate | 0.874 | 0.900 | 0.887 | 100 |
|  |  |  |  |  |
| Accuracy | - | - | 0.827 | 300 |
| Macro avg | 0.826 | 0.827 | 0.822 | 300 |
| Weighted avg | 0.826 | 0.827 | 0.822 | 300 |

| **Table S20. results on 2nd round, Gemma-2-9b-it (finetune), random seed=2** | | | | |
| --- | --- | --- | --- | --- |
|  | Precision | Recall | F1-score | Support |
| Unclear | 0.767 | 0.690 | 0.726 | 100 |
| Opposite | 0.810 | 0.850 | 0.829 | 100 |
| Advocate | 0.829 | 0.870 | 0.849 | 100 |
|  |  |  |  |  |
| Accuracy | - | - | 0.803 | 300 |
| Macro avg | 0.802 | 0.803 | 0.801 | 300 |
| Weighted avg | 0.802 | 0.803 | 0.801 | 300 |

| **Table S21. results on 3rd round, Gemma-2-9b-it (finetune), random seed=3** | | | | |
| --- | --- | --- | --- | --- |
|  | Precision | Recall | F1-score | Support |
| Unclear | 0.870 | 0.470 | 0.610 | 100 |
| Opposite | 0.777 | 0.940 | 0.851 | 100 |
| Advocate | 0.744 | 0.930 | 0.827 | 100 |
|  |  |  |  |  |
| Accuracy | - | - | 0.780 | 300 |
| Macro avg | 0.797 | 0.780 | 0.763 | 300 |
| Weighted avg | 0.797 | 0.780 | 0.763 | 300 |

| **Table S22. results on 1st round, llama-3.1-8b-it (finetune), random seed=1** | | | | |
| --- | --- | --- | --- | --- |
|  | Precision | Recall | F1-score | Support |
| Unclear | 0.413 | 1.000 | 0.602 | 100 |
| Opposite | 1.000 | 0.170 | 0.291 | 100 |
| Advocate | 0.980 | 0.500 | 0.662 | 100 |
|  |  |  |  |  |
| Accuracy | - | - | 0.557 | 300 |
| Macro avg | 0.804 | 0.557 | 0.518 | 300 |
| Weighted avg | 0.804 | 0.557 | 0.518 | 300 |

| **Table S23. results on 2nd round, llama-3.1-8b-it (finetune), random seed=2** | | | | |
| --- | --- | --- | --- | --- |
|  | Precision | Recall | F1-score | Support |
| Unclear | 0.429 | 0.970 | 0.595 | 100 |
| Opposite | 1.000 | 0.170 | 0.291 | 100 |
| Advocate | 0.930 | 0.530 | 0.675 | 100 |
|  |  |  |  |  |
| Accuracy | - | - | 0.557 | 300 |
| Macro avg | 0.786 | 0.557 | 0.520 | 300 |
| Weighted avg | 0.786 | 0.557 | 0.520 | 300 |

| **Table S24. results on 3rd round, llama-3.1-8b-it (finetune), random seed=3** | | | | |
| --- | --- | --- | --- | --- |
|  | Precision | Recall | F1-score | Support |
| Unclear | 0..436 | 0.960 | 0.600 | 100 |
| Opposite | 0.960 | 0.240 | 0.384 | 100 |
| Advocate | 0.927 | 0.510 | 0.658 | 100 |
|  |  |  |  |  |
| Accuracy | - | - | 0.570 | 300 |
| Macro avg | 0.775 | 0.570 | 0.547 | 300 |
| Weighted avg | 0.775 | 0.570 | 0.547 | 300 |

## Results on Gemini 1.0 pro

All baseline or finetuned models were tested three times, with 100 test data randomly selected from each stance. 166 tweets from each stance were reelected for finetuning.

| **Table S25. results on 1st round, Gemini 1.0 pro (baseline), random seed=1** | | | | |
| --- | --- | --- | --- | --- |
|  | Precision | Recall | F1-score | Support |
| Unclear | 0.556 | 0.990 | 0.712 | 100 |
| Opposite | 0.985 | 0.640 | 0.776 | 100 |
| Advocate | 1.000 | 0.570 | 0.726 | 100 |
|  |  |  |  |  |
| Accuracy | - | - | 0.733 | 300 |
| Macro avg | 0.847 | 0.733 | 0.738 | 300 |
| Weighted avg | 0.847 | 0.733 | 0.738 | 300 |

| **Table S26. results on 2nd round, Gemini 1.0 pro (baseline), random seed=2** | | | | |
| --- | --- | --- | --- | --- |
|  | Precision | Recall | F1-score | Support |
| Unclear | 0.503 | 0.950 | 0.657 | 100 |
| Opposite | 0.870 | 0.600 | 0.710 | 100 |
| Advocate | 0.976 | 0.410 | 0.577 | 100 |
|  |  |  |  |  |
| Accuracy | - | - | 0.653 | 300 |
| Macro avg | 0.783 | 0.653 | 0.648 | 300 |
| Weighted avg | 0.783 | 0.653 | 0.648 | 300 |

| **Table S27. results on 3rd round, Gemini 1.0 pro (baseline), random seed=3** | | | | |
| --- | --- | --- | --- | --- |
|  | Precision | Recall | F1-score | Support |
| Unclear | 0.530 | 0.980 | 0.688 | 100 |
| Opposite | 0.968 | 0.600 | 0.741 | 100 |
| Advocate | 0.981 | 0.520 | 0.680 | 100 |
|  |  |  |  |  |
| Accuracy | - | - | 0.700 | 300 |
| Macro avg | 0.826 | 0.700 | 0.703 | 300 |
| Weighted avg | 0.826 | 0.700 | 0.703 | 300 |

| **Table S28. results on 1st round, Gemini 1.0 pro (finetune), random seed=1** | | | | |
| --- | --- | --- | --- | --- |
|  | Precision | Recall | F1-score | Support |
| Unclear | 0.933 | 0.84 | 0.884 | 100 |
| Opposite | 0.906 | 0.96 | 0.932 | 100 |
| Advocate | 0.913 | 0.94 | 0.926 | 100 |
|  |  |  |  |  |
| Accuracy | - | - | 0.913 | 300 |
| Macro avg | 0.688 | 0.685 | 0.686 | 300 |
| Weighted avg | 0.917 | 0.913 | 0.914 | 300 |

| **Table S29. results on 2nd round, Gemini 1.0 pro (finetune), random seed=2** | | | | |
| --- | --- | --- | --- | --- |
|  | Precision | Recall | F1-score | Support |
| Unclear | 0.906 | 0.77 | 0.832 | 100 |
| Opposite | 0.856 | 0.95 | 0.9 | 100 |
| Advocate | 0.922 | 0.94 | 0.931 | 100 |
|  |  |  |  |  |
| Accuracy | - | - | 0.887 | 300 |
| Macro avg | 0.671 | 0.665 | 0.666 | 300 |
| Weighted avg | 0.894 | 0.887 | 0.888 | 300 |

| **Table S30. results on 3rd round, Gemini 1.0 pro (finetune), random seed=3** | | | | |
| --- | --- | --- | --- | --- |
|  | Precision | Recall | F1-score | Support |
| Unclear | 0.892 | 0.74 | 0.809 | 100 |
| Opposite | 0.87 | 0.94 | 0.904 | 100 |
| Advocate | 0.87 | 0.94 | 0.904 | 100 |
|  |  |  |  |  |
| Accuracy | - | - | 0.873 | 300 |
| Macro avg | 0.658 | 0.655 | 0.654 | 300 |
| Weighted avg | 0.877 | 0.873 | 0.872 | 300 |
