# Supplementary material for: Understanding Human Papillomavirus Vaccination Hesitancy in Japan Using Social Media: Content Analysis
Source: J Med Internet Res. 2025 Feb 11;27:e68881. doi: 10.2196/68881 (PMC11862774; doi:10.2196/68881)
Supplement: Multimedia Appendix 6 [file jmir_v27i1e68881_app6.docx]

### F1 score of Gemini 1.0 pro trained with different ratios of labels

| **Table S1. F1 score of models trained with data at different ratios of unclear, opposite, and advocate** | | | | | | | |
| --- | --- | --- | --- | --- | --- | --- | --- |
| Round | Unclear: Opposite: Advocate | | | | | | |
|  | 166:166:166 | 150:200:150 | 125:250:125 | 100:300:100 | 75:350:75 | 50:400:50 | 25:450:25 |
| 1st | 0.914 | 0.955 | 0.930 | 0.948 | 0.920 | 0.952 | 0.887 |
| 2nd | 0.888 | 0.970 | 0.967 | 0.957 | 0.950 | 0.923 | 0.926 |
| 3rd | 0.872 | 0.978 | 0.958 | 0.968 | 0.961 | 0.945 | 0.893 |
| Average | 0.891 | **0.968** | 0.952 | 0.958 | 0.944 | 0.940 | 0.902 |

### Details results of training data ratio selection

| **Table S2. Ratio selection 1st round, unclear:opposite:advocate=166:166:166, random seed=1** | | | | |
| --- | --- | --- | --- | --- |
|  | Precision | Recall | F1-score | Support |
| Unclear | 0.933 | 0.84 | 0.884 | 100 |
| Opposite | 0.906 | 0.96 | 0.932 | 100 |
| Advocate | 0.913 | 0.94 | 0.926 | 100 |
|  |  |  |  |  |
| Accuracy | - | - | 0.913 | 300 |
| Macro avg | 0.688 | 0.685 | 0.686 | 300 |
| Weighted avg | 0.917 | 0.913 | 0.914 | 300 |

| **Table S3. Ratio selection 1st round, unclear:opposite:advocate=150:200:150, random seed=1** | | | | |
| --- | --- | --- | --- | --- |
|  | Precision | Recall | F1-score | Support |
| Unclear | 0.949 | 0.93 | 0.939 | 100 |
| Opposite | 0.97 | 0.97 | 0.97 | 100 |
| Advocate | 0.95 | 0.96 | 0.955 | 100 |
|  |  |  |  |  |
| Accuracy | - | - | 0.953 | 300 |
| Macro avg | 0.717 | 0.715 | 0.716 | 300 |
| Weighted avg | 0.956 | 0.953 | 0.955 | 300 |

| **Table S4. Ratio selection 1st round, unclear:opposite:advocate=125:250:125, random seed=1** | | | | |
| --- | --- | --- | --- | --- |
|  | Precision | Recall | F1-score | Support |
| Unclear | 0.988 | 0.81 | 0.89 | 100 |
| Opposite | 0.9 | 0.99 | 0.943 | 100 |
| Advocate | 0.925 | 0.99 | 0.957 | 100 |
|  |  |  |  |  |
| Accuracy | - | - | 0.93 | 300 |
| Macro avg | 0.703 | 0.698 | 0.697 | 300 |
| Weighted avg | 0.938 | 0.93 | 0.93 | 300 |

| **Table S5. Ratio selection 1st round, unclear:opposite:advocate=100:300:100, random seed=1** | | | | |
| --- | --- | --- | --- | --- |
|  | Precision | Recall | F1-score | Support |
| Unclear | 0.947 | 0.9 | 0.923 | 100 |
| Opposite | 0.917 | 0.99 | 0.952 | 100 |
| Advocate | 0.99 | 0.95 | 0.969 | 100 |
|  |  |  |  |  |
| Accuracy | - | - | 0.947 | 300 |
| Macro avg | 0.713 | 0.71 | 0.711 | 300 |
| Weighted avg | 0.951 | 0.947 | 0.948 | 300 |

| **Table S6. Ratio selection 1st round, unclear:opposite:advocate=75:350:75, random seed=1** | | | | |
| --- | --- | --- | --- | --- |
|  | Precision | Recall | F1-score | Support |
| Unclear | 0.964 | 0.8 | 0.874 | 100 |
| Opposite | 0.876 | 0.99 | 0.93 | 100 |
| Advocate | 0.942 | 0.97 | 0.956 | 100 |
|  |  |  |  |  |
| Accuracy | - | - | 0.92 | 300 |
| Macro avg | 0.695 | 0.69 | 0.69 | 300 |
| Weighted avg | 0.927 | 0.92 | 0.92 | 300 |

| **Table S7. Ratio selection 1st round, unclear:opposite:advocate=50:400:50, random seed=1** | | | | |
| --- | --- | --- | --- | --- |
|  | Precision | Recall | F1-score | Support |
| Unclear | 0.93 | 0.93 | 0.93 | 100 |
| Opposite | 0.934 | 0.99 | 0.961 | 100 |
| Advocate | 1 | 0.93 | 0.964 | 100 |
|  |  |  |  |  |
| Accuracy | - | - | 0.95 | 300 |
| Macro avg | 0.716 | 0.713 | 0.714 | 300 |
| Weighted avg | 0.955 | 0.95 | 0.952 | 300 |

| **Table S8. Ratio selection 1st round, unclear:opposite:advocate=25:450:25, random seed=1** | | | | |
| --- | --- | --- | --- | --- |
|  | Precision | Recall | F1-score | Support |
| Unclear | 1 | 0.68 | 0.81 | 100 |
| Opposite | 0.792 | 0.99 | 0.88 | 100 |
| Advocate | 0.943 | 1 | 0.971 | 100 |
|  |  |  |  |  |
| Accuracy | - | - | 0.89 | 300 |
| Macro avg | 0.684 | 0.667 | 0.665 | 300 |
| Weighted avg | 0.912 | 0.89 | 0.887 | 300 |

| **Table S9. Ratio selection 2nd round, unclear:opposite:advocate=166:166:166, random seed=2** | | | | |
| --- | --- | --- | --- | --- |
|  | Precision | Recall | F1-score | Support |
| Unclear | 0.906 | 0.77 | 0.832 | 100 |
| Opposite | 0.856 | 0.95 | 0.9 | 100 |
| Advocate | 0.922 | 0.94 | 0.931 | 100 |
|  |  |  |  |  |
| Accuracy | - | - | 0.887 | 300 |
| Macro avg | 0.671 | 0.665 | 0.666 | 300 |
| Weighted avg | 0.894 | 0.887 | 0.888 | 300 |

| **Table S10. Ratio selection 2nd round, unclear:opposite:advocate=150:200:150, random seed=2** | | | | |
| --- | --- | --- | --- | --- |
|  | Precision | Recall | F1-score | Support |
| Unclear | 0.95 | 0.96 | 0.955 | 100 |
| Opposite | 0.98 | 0.97 | 0.975 | 100 |
| Advocate | 0.99 | 0.97 | 0.98 | 100 |
|  |  |  |  |  |
| Accuracy | - | - | 0.967 | 300 |
| Macro avg | 0.73 | 0.725 | 0.727 | 300 |
| Weighted avg | 0.973 | 0.967 | 0.97 | 300 |

| **Ratio selection 2nd round, unclear:opposite:advocate=125:250:125, random seed=2** | | | | |
| --- | --- | --- | --- | --- |
|  | Precision | Recall | F1-score | Support |
| Unclear | 0.95 | 0.95 | 0.95 | 100 |
| Opposite | 0.98 | 0.97 | 0.975 | 100 |
| Advocate | 0.98 | 0.97 | 0.975 | 100 |
|  |  |  |  |  |
| Accuracy | - | - | 0.963 | 300 |
| Macro avg | 0.727 | 0.722 | 0.725 | 300 |
| Weighted avg | 0.97 | 0.963 | 0.967 | 300 |

| **Ratio selection 2nd round, unclear:opposite:advocate=100:300:100, random seed=2** | | | | |
| --- | --- | --- | --- | --- |
|  | Precision | Recall | F1-score | Support |
| Unclear | 0.907 | 0.98 | 0.942 | 100 |
| Opposite | 0.99 | 0.95 | 0.969 | 100 |
| Advocate | 0.989 | 0.93 | 0.959 | 100 |
|  |  |  |  |  |
| Accuracy | - | - | 0.953 | 300 |
| Macro avg | 0.722 | 0.715 | 0.718 | 300 |
| Weighted avg | 0.962 | 0.953 | 0.957 | 300 |

| **Ratio selection 2nd round, unclear:opposite:advocate=75:350:75, random seed=2** | | | | |
| --- | --- | --- | --- | --- |
|  | Precision | Recall | F1-score | Support |
| Unclear | 0.905 | 0.95 | 0.927 | 100 |
| Opposite | 0.961 | 0.98 | 0.97 | 100 |
| Advocate | 1 | 0.91 | 0.953 | 100 |
|  |  |  |  |  |
| Accuracy | - | - | 0.947 | 300 |
| Macro avg | 0.716 | 0.71 | 0.713 | 300 |
| Weighted avg | 0.955 | 0.947 | 0.95 | 300 |

| **Ratio selection 2nd round, unclear:opposite:advocate=50:400:50, random seed=2** | | | | |
| --- | --- | --- | --- | --- |
|  | Precision | Recall | F1-score | Support |
| Unclear | 0.841 | 0.95 | 0.892 | 100 |
| Opposite | 0.961 | 0.98 | 0.97 | 100 |
| Advocate | 1 | 0.83 | 0.907 | 100 |
|  |  |  |  |  |
| Accuracy | - | - | 0.92 | 300 |
| Macro avg | 0.7 | 0.69 | 0.692 | 300 |
| Weighted avg | 0.934 | 0.92 | 0.923 | 300 |

| **Ratio selection 2nd round, unclear:opposite:advocate=25:450:25, random seed=2** | | | | |
| --- | --- | --- | --- | --- |
|  | Precision | Recall | F1-score | Support |
| Unclear | 0.944 | 0.84 | 0.889 | 100 |
| Opposite | 0.861 | 0.99 | 0.921 | 100 |
| Advocate | 1 | 0.94 | 0.969 | 100 |
|  |  |  |  |  |
| Accuracy | - | - | 0.923 | 300 |
| Macro avg | 0.701 | 0.693 | 0.695 | 300 |
| Weighted avg | 0.935 | 0.923 | 0.926 | 300 |

| **Ratio selection 3rd round, unclear:opposite:advocate=166:166:166, random seed=3** | | | | |
| --- | --- | --- | --- | --- |
|  | Precision | Recall | F1-score | Support |
| Unclear | 0.892 | 0.74 | 0.809 | 100 |
| Opposite | 0.87 | 0.94 | 0.904 | 100 |
| Advocate | 0.87 | 0.94 | 0.904 | 100 |
|  |  |  |  |  |
| Accuracy | - | - | 0.873 | 300 |
| Macro avg | 0.658 | 0.655 | 0.654 | 300 |
| Weighted avg | 0.877 | 0.873 | 0.872 | 300 |

| **Ratio selection 3rd round, unclear:opposite:advocate=150:200:150, random seed=3** | | | | |
| --- | --- | --- | --- | --- |
|  | Precision | Recall | F1-score | Support |
| Unclear | 0.98 | 0.96 | 0.97 | 100 |
| Opposite | 0.98 | 1 | 0.99 | 100 |
| Advocate | 0.98 | 0.97 | 0.975 | 100 |
|  |  |  |  |  |
| Accuracy | - | - | 0.977 | 300 |
| Macro avg | 0.735 | 0.732 | 0.734 | 300 |
| Weighted avg | 0.98 | 0.977 | 0.978 | 300 |

| **Ratio selection 3rd round, unclear:opposite:advocate=125:250:125, random seed=3** | | | | |
| --- | --- | --- | --- | --- |
|  | Precision | Recall | F1-score | Support |
| Unclear | 0.949 | 0.93 | 0.939 | 100 |
| Opposite | 0.952 | 0.99 | 0.971 | 100 |
| Advocate | 0.979 | 0.95 | 0.964 | 100 |
|  |  |  |  |  |
| Accuracy | - | - | 0.957 | 300 |
| Macro avg | 0.72 | 0.718 | 0.719 | 300 |
| Weighted avg | 0.96 | 0.957 | 0.958 | 300 |

| **Ratio selection 3rd round, unclear:opposite:advocate=100:300:100, random seed=3** | | | | |
| --- | --- | --- | --- | --- |
|  | Precision | Recall | F1-score | Support |
| Unclear | 1 | 0.9 | 0.947 | 100 |
| Opposite | 0.962 | 1 | 0.98 | 100 |
| Advocate | 0.952 | 1 | 0.976 | 100 |
|  |  |  |  |  |
| Accuracy | - | - | 0.967 | 300 |
| Macro avg | 0.728 | 0.725 | 0.726 | 300 |
| Weighted avg | 0.971 | 0.967 | 0.968 | 300 |

| **Ratio selection 3rd round, unclear:opposite:advocate=75:350:75, random seed=3** | | | | |
| --- | --- | --- | --- | --- |
|  | Precision | Recall | F1-score | Support |
| Unclear | 1 | 0.89 | 0.942 | 100 |
| Opposite | 0.917 | 1 | 0.957 | 100 |
| Advocate | 0.98 | 0.99 | 0.985 | 100 |
|  |  |  |  |  |
| Accuracy | - | - | 0.96 | 300 |
| Macro avg | 0.724 | 0.72 | 0.721 | 300 |
| Weighted avg | 0.966 | 0.96 | 0.961 | 300 |

| **Ratio selection 3rd round, unclear:opposite:advocate=50:400:50, random seed=3** | | | | |
| --- | --- | --- | --- | --- |
|  | Precision | Recall | F1-score | Support |
| Unclear | 0.919 | 0.91 | 0.915 | 100 |
| Opposite | 0.926 | 1 | 0.962 | 100 |
| Advocate | 1 | 0.92 | 0.958 | 100 |
|  |  |  |  |  |
| Accuracy | - | - | 0.943 | 300 |
| Macro avg | 0.711 | 0.708 | 0.709 | 300 |
| Weighted avg | 0.948 | 0.943 | 0.945 | 300 |

| **Ratio selection 3rd round, unclear:opposite:advocate=25:450:25, random seed=3** | | | | |
| --- | --- | --- | --- | --- |
|  | Precision | Recall | F1-score | Support |
| Unclear | 0.986 | 0.7 | 0.819 | 100 |
| Opposite | 0.826 | 1 | 0.905 | 100 |
| Advocate | 0.925 | 0.99 | 0.957 | 100 |
|  |  |  |  |  |
| Accuracy | - | - | 0.897 | 300 |
| Macro avg | 0.684 | 0.672 | 0.67 | 300 |
| Weighted avg | 0.913 | 0.897 | 0.893 | 300 |
