# Supplementary material for: Understanding Human Papillomavirus Vaccination Hesitancy in Japan Using Social Media: Content Analysis
Source: J Med Internet Res. 2025 Feb 11;27:e68881. doi: 10.2196/68881 (PMC11862774; doi:10.2196/68881)
Supplement: Multimedia Appendix 8 [file jmir_v27i1e68881_app8.docx]

Table S1. First-round model performance by category and tier, random seed=1

| **Overall** | | | | |
| --- | --- | --- | --- | --- |
|  | Precision | Recall | F1-score | Support |
| Unclear | 0.956 | 0.855 | 0.903 | 3265 |
| Opposite | 0.641 | 0.972 | 0.772 | 497 |
| Advocate | 0.866 | 0.914 | 0.889 | 1496 |
|  |  |  |  |  |
| Accuracy | - | - | 0.883 | 5258 |
| Macro avg | 0.493 | 0.548 | 0.513 | 5258 |
| Weighted avg | 0.901 | 0.883 | 0.887 | 5258 |
| **Tier1** | | | | |
| Unclear | 0.978 | 0.919 | 0.947 | 2904 |
| Opposite | 0.697 | 0.989 | 0.818 | 356 |
| Advocate | 0.937 | 0.953 | 0.945 | 1290 |
|  |  |  |  |  |
| Accuracy | - | - | 0.934 | 4550 |
| Macro avg | 0.522 | 0.572 | 0.542 | 4550 |
| Weighted avg | 0.944 | 0.934 | 0.936 | 4550 |
| **Tier2** | | | | |
| Unclear | 0.649 | 0.350 | 0.455 | 354 |
| Opposite | 0.535 | 0.929 | 0.679 | 141 |
| Advocate | 0.525 | 0.675 | 0.590 | 206 |
|  |  |  |  |  |
| Accuracy | - | - | 0.562 | 701 |
| Macro avg | 0.569 | 0.651 | 0.575 | 701 |
| Weighted avg | 0.590 | 0.562 | 0.540 | 701 |
| **Tier3** | | | | |
| Unclear | 0 | 0 | 0 | 7 |
| Opposite | 0 | 0 | 0 | 0 |
| Advocate | 0 | 0 | 0 | 0 |
|  |  |  |  |  |
| Accuracy | - | - | 0 | 7 |
| Macro avg | 0 | 0 | 0 | 7 |
| Weighted avg | 0 | 0 | 0 | 7 |

Table S2. Second-round model performance by category and tier, random seed=2

| **Overall** | | | | |
| --- | --- | --- | --- | --- |
|  | Precision | Recall | F1-score | Support |
| Unclear | 0.905 | 0.966 | 0.935 | 3265 |
| Opposite | 0.833 | 0.851 | 0.842 | 497 |
| Advocate | 0.97 | 0.816 | 0.886 | 1496 |
|  |  |  |  |  |
| Accuracy | - | - | 0.913 | 5258 |
| Macro avg | 0.677 | 0.658 | 0.666 | 5258 |
| Weighted avg | 0.917 | 0.913 | **0.912** | 5258 |
| **Tier1** | | | | |
| Unclear | 0.952 | 0.986 | 0.969 | 2904 |
| Opposite | 0.911 | 0.947 | 0.928 | 356 |
| Advocate | 0.991 | 0.895 | 0.941 | 1290 |
|  |  |  |  |  |
| Accuracy | - | - | 0.957 | 4550 |
| Macro avg | 0.713 | 0.707 | 0.709 | 4550 |
| Weighted avg | 0.96 | 0.957 | 0.958 | 4550 |
| **Tier2** | | | | |
| Unclear | 0.609 | 0.819 | 0.699 | 354 |
| Opposite | 0.642 | 0.610 | 0.625 | 141 |
| Advocate | 0.725 | 0.32 | 0.444 | 206 |
|  |  |  |  |  |
| Accuracy | - | - | 0.631 | 701 |
| Macro avg | 0.659 | 0.583 | 0.59 | 701 |
| Weighted avg | 0.65 | 0.631 | 0.609 | 701 |
| **Tier3** | | | | |
| Unclear | 1 | 0.143 | 0.25 | 7 |
| Opposite | 0 | 0 | 0 | 0 |
| Advocate | 0 | 0 | 0 | 0 |
|  |  |  |  |  |
| Accuracy | - | - | 0.143 | 7 |
| Macro avg | 0.333 | 0.048 | 0.083 | 7 |
| Weighted avg | 1 | 0.143 | 0.25 | 7 |

Table S3. Third-round model performance by category and tier, random seed=3

| **Overall** | | | | |
| --- | --- | --- | --- | --- |
|  | Precision | Recall | F1-score | Support |
| Unclear | 0.897 | 0.967 | 0.93 | 3265 |
| Opposite | 0.854 | 0.849 | 0.852 | 497 |
| Advocate | 0.968 | 0.801 | 0.877 | 1496 |
|  |  |  |  |  |
| Accuracy | - | - | 0.909 | 5258 |
| Macro avg | 0.68 | 0.654 | 0.665 | 5258 |
| Weighted avg | 0.913 | 0.909 | 0.908 | 5258 |
| **Tier1** | | | | |
| Unclear | 0.946 | 0.985 | 0.965 | 2904 |
| Opposite | 0.921 | 0.952 | 0.936 | 356 |
| Advocate | 0.990 | 0.885 | 0.935 | 1290 |
|  |  |  |  |  |
| Accuracy | - | - | 0.954 | 4550 |
| Macro avg | 0.714 | 0.706 | 0.709 | 4550 |
| Weighted avg | 0.957 | 0.954 | 0.954 | 4550 |
| **Tier2** | | | | |
| Unclear | 0.59 | 0.816 | 0.685 | 354 |
| Opposite | 0.664 | 0.589 | 0.624 | 141 |
| Advocate | 0.663 | 0.277 | 0.39 | 206 |
|  |  |  |  |  |
| Accuracy | - | - | 0.612 | 701 |
| Macro avg | 0.639 | 0.561 | 0.566 | 701 |
| Weighted avg | 0.626 | 0.612 | 0.586 | 701 |
| **Tier3** | | | | |
| Unclear | 1 | 0.857 | 0.923 | 7 |
| Opposite | 0 | 0 | 0 | 0 |
| Advocate | - | - | - | - |
|  |  |  |  |  |
| Accuracy | - | - | 0.857 | 7 |
| Macro avg | 0.5 | 0.429 | 0.462 | 7 |
| Weighted avg | 1 | 0.857 | 0.923 | 7 |

Table S4. Fourth-round model performance by category and tier, random seed=4

| **Overall** | | | | |
| --- | --- | --- | --- | --- |
|  | Precision | Recall | F1-score | Support |
| Unclear | 0.941 | 0.963 | 0.952 | 3265 |
| Opposite | 0.867 | 0.855 | 0.861 | 497 |
| Advocate | 0.954 | 0.906 | 0.929 | 1496 |
|  |  |  |  |  |
| Accuracy | - | - | 0.936 | 5258 |
| Macro avg | 0.69 | 0.681 | 0.686 | 5258 |
| Weighted avg | 0.937 | 0.936 | **0.937** | 5258 |
| **Tier1** | | | | |
| Unclear | 0.982 | 0.988 | 0.985 | 2904 |
| Opposite | 0.937 | 0.963 | 0.950 | 356 |
| Advocate | 0.991 | 0.964 | 0.978 | 1290 |
|  |  |  |  |  |
| Accuracy | - | - | 0.980 | 4550 |
| Macro avg | 0.727 | 0.729 | 0.728 | 4550 |
| Weighted avg | 0.981 | 0.980 | 0.980 | 4550 |
| **Tier2** | | | | |
| Unclear | 0.650 | 0.757 | 0.7 | 354 |
| Opposite | 0.672 | 0.582 | 0.624 | 141 |
| Advocate | 0.671 | 0.544 | 0.601 | 206 |
|  |  |  |  |  |
| Accuracy | - | - | 0.659 | 701 |
| Macro avg | 0.664 | 0.627 | 0.641 | 701 |
| Weighted avg | 0.661 | 0.659 | 0.655 | 701 |
| **Tier3** | | | | |
| Unclear | 1 | 0.714 | 0.833 | 7 |
| Opposite | 0 | 0 | 0 | 0 |
| Advocate | - | - | - | - |
|  |  |  |  |  |
| Accuracy | - | - | 0.714 | 7 |
| Macro avg | 0.5 | 0.357 | 0.417 | 7 |
| Weighted avg | 1 | 0.714 | 0.833 | 7 |

Table S5. Fifth-round model performance by category and tier, random seed=5

| **Overall** | | | | |
| --- | --- | --- | --- | --- |
|  | Precision | Recall | F1-score | Support |
| Unclear | 0.979 | 0.899 | 0.937 | 3265 |
| Opposite | 0.783 | 0.932 | 0.851 | 497 |
| Advocate | 0.875 | 0.972 | 0.921 | 1496 |
|  |  |  |  |  |
| Accuracy | - | - | 0.923 | 5258 |
| Macro avg | 0.659 | 0.701 | 0.677 | 5258 |
| Weighted avg | 0.931 | 0.923 | **0.924** | 5258 |
| **Tier1** | | | | |
| Unclear | 0.995 | 0.956 | 0.975 | 2904 |
| Opposite | 0.884 | 0.983 | 0.931 | 356 |
| Advocate | 0.940 | 0.991 | 0.965 | 1290 |
|  |  |  |  |  |
| Accuracy | - | - | 0.968 | 4550 |
| Macro avg | 0.705 | 0.733 | 0.718 | 4550 |
| Weighted avg | 0.971 | 0.968 | 0.969 | 4550 |
| **Tier2** | | | | |
| Unclear | 0.758 | 0.452 | 0.566 | 354 |
| Opposite | 0.592 | 0.801 | 0.681 | 141 |
| Advocate | 0.585 | 0.85 | 0.693 | 206 |
|  |  |  |  |  |
| Accuracy | - | - | 0.639 | 701 |
| Macro avg | 0.645 | 0.701 | 0.647 | 701 |
| Weighted avg | 0.674 | 0.639 | 0.627 | 701 |
| **Tier3** | | | | |
| Unclear | 0 | 0 | 0 | 7 |
| Opposite | 0 | 0 | 0 | 0 |
| Advocate | 0 | 0 | 0 | 0 |
|  |  |  |  |  |
| Accuracy | - | - | 0 | 7 |
| Macro avg | 0 | 0 | 0 | 7 |
| Weighted avg | 0 | 0 | 0 | 7 |
